# Longitudinal SOFA score trajectories and risk stratification in ICU patients with *Staphylococcus aureus* bloodstream infection: insights from group-based trajectory modeling

**DOI:** 10.3389/fcimb.2026.1756348

**Published:** 2026-02-09

**Authors:** Heyu Chen, Yushi Fan, Ruomeng Hu, Qing Yu, Jiafei Yu, Xinyun Zhang, Hongwei Zhou, Kai Zhang, Wei Cui, Shufang Zhang, Li Zhong, Haoliang Qian, Gensheng Zhang

**Affiliations:** 1 Department of Critical Care Medicine, Second Affiliated Hospital, Zhejiang University School of Medicine, Hangzhou, Zhejiang, China; 2 Department of Critical Care Medicine, Haiyan People’s Hospital, Jiaxing, Zhejiang, China; 3 Department of Clinical Microbiology Laboratory, Second Affiliated Hospital, Zhejiang University School of Medicine, Hangzhou, Zhejiang, China; 4 Department of Cardiology, The Second Affiliated Hospital, Zhejiang University School of Medicine, Hangzhou, Zhejiang, China; 5 State Key Laboratory of Transvascular Implantation Devices, Heart Regeneration and Repair Key Laboratory of Zhejiang Province, Hangzhou, Zhejiang, China; 6 Department of Critical Care Medicine, The First People’s Hospital of Huzhou, First Affiliated Hospital of Huzhou University, Huzhou, Zhejiang, China; 7 Zhejiang University - University of Illinois Urbana-Champaign (ZJU-UIUC) Institute, Interdisciplinary Center for Quantum Information, State Key Laboratory of Extreme Photonics and Instrumentation, ZJU-Hangzhou Global Science and Technology Innovation Center, Hangzhou, Zhejiang, China; 8 Key Lab. of Advanced Micro/Nano Electronic Devices & Smart Systems of Zhejiang, Zhejiang University, Hangzhou, Zhejiang, China; 9 Key Laboratory of Multiple Organ Failure (Zhejiang University), Ministry of Education, Hangzhou, Zhejiang, China

**Keywords:** group-based trajectory modeling (GBTM), intensive care unit (ICU), machine learning, multiple-organ dysfunction, sequential organ failure assessment score (SOFA score), *Staphylococcus aureus* bloodstream infection

## Abstract

**Background:**

*Staphylococcus aureus* bloodstream infection (SA-BSI) is a life-threatening condition in ICU patients, often leading to progressive multi-organ dysfunction. Traditional static assessments may underestimate the dynamic nature of organ failure. We aimed to identify distinct organ dysfunction trajectories and evaluate their prognostic significance using a data-driven and interpretable machine learning approach.

**Methods:**

ICU patients with SA-BSI from two independent cohorts admitted between 2008 and 2024 were retrospectively analyzed (MIMIC-IV, n=834; the Second Affiliated Hospital of Zhejiang University School of Medicine (SAHZU), n=151). Daily Sequential Organ Failure Assessment (SOFA) scores from Day -1 to +3 were used to derive trajectory subgroups via group-based trajectory modeling. Associations with in-hospital mortality were assessed using multivariable Cox regression and Kaplan-Meier analysis. An XGBoost model was developed to predict trajectory group membership based on baseline ICU admission variables, with interpretability assessed via SHAP values.

**Results:**

Three reproducible SOFA trajectory groups were identified in both cohorts, representing stable, moderately worsening, and severely deteriorating clinical courses. Compared with the stable group, patients in the severely deteriorating group had a markedly increased risk of in-hospital mortality (HR 4.60, 95% CI 3.49–6.07), with consistent effects observed across both cohorts. The XGBoost model demonstrated strong predictive performance for identifying severely deteriorating trajectories (AUC 0.96), and SHAP analysis revealed biologically coherent predictors underlying each trajectory.

**Conclusions:**

Early ICU data can predict dynamic organ dysfunction trajectories in SA-BSI patients. Trajectory-based phenotyping, combined with interpretable machine learning, offers a clinically valuable framework for early risk stratification and individualized ICU management.

## Introduction

*Staphylococcus aureus* (*S. aureus*) is a major human pathogen responsible for a wide spectrum of diseases, from superficial skin infections to life-threatening sepsis (Maurer et al., 2015). It is the leading cause of fatal bloodstream infections (BSIs), with mortality rates ranging from 20% to 40%, posing a significant public health burden worldwide (Claes et al., 2014; Cooper et al., 2020; Krasaewes et al., 2022). In intensive care unit (ICU) patients, *S. aureus* bloodstream infection (SA-BSI) is particularly challenging due to host immunosuppression, frequent invasive procedures, and multiple comorbidities. These factors contribute to prolonged length of stay (LOS) in hospital and ICU, elevated resource utilization, and markedly higher mortality compared to general ward populations (Vincent et al., 2009). While substantial research has centered on Gram-negative bacteremia, especially in regions like China, SA-BSI remains comparatively under-investigated, particularly regarding its dynamic clinical evolution and risk stratification in ICU settings. As the global population ages and antimicrobial resistance escalates, early recognition and individualized management of SA-BSI are increasingly vital to improving outcomes in this vulnerable population.

The Sequential Organ Failure Assessment (SOFA) score quantifies dysfunction across six organ systems—respiratory, coagulation, hepatic, renal, cardiovascular, and central nervous system (CNS)—on a 0–4 scale, with higher scores indicating more severe organ failure (Vincent et al., 1996). It has long been used as a prognostic tool in critically ill patients (Vincent et al., 1998). However, conventional single-time-point SOFA measurements offer only static snapshots of a patient’s condition and may fail to reflect the dynamic and often nonlinear progression of organ dysfunction (OD) in the ICU (Yang et al., 2022). Given the rapid physiological shifts common in critical illness, a longitudinal assessment of SOFA trajectories could provide deeper insight into disease evolution, capturing both subclinical deterioration and unexpected recovery. These temporal trends are particularly valuable for early risk stratification and timely therapeutic decision-making. In the context of SA-BSI, where delays in diagnosis and intervention can be fatal, tracking OD progression over time may offer critical prognostic information and inform personalized management strategies.

Group-based trajectory modeling (GBTM), originally proposed by Nagin (2005), is a statistical method for identifying latent subgroups within a population that share similar longitudinal trends. While first introduced in criminology, GBTM has gained traction in clinical research, where time-series data, such as serial SOFA scores, are increasingly available (Nagin and Odgers, 2010). By enabling data-driven phenotyping based on the evolution of clinical parameters, GBTM offers a promising framework for uncovering disease heterogeneity in the ICU (Wang et al., 2024).

Previous studies have applied GBTM or similar approaches to analyze SOFA trajectories in patients with sepsis (Xu et al., 2022), COVID-19 (Yu et al., 2024), and other critical illnesses, and to investigate temporal trends of inflammatory markers (e.g., C-reactive protein [CRP]) and vital signs in BSI (Gu et al., 2024). However, no prior research has specifically focused on longitudinal SOFA score trajectories in ICU patients with SA-BSI. This represents a critical knowledge gap, particularly given the dynamic and multisystem nature of OD in this population. Notably, the early phase of BSI, which is before microbiological confirmation, is a high-stakes window in which empirical treatment decisions must be made. During this period, early signs of multi-organ dysfunction (MOD) may be subtle or misinterpreted, and prognostically relevant trends may be overlooked. Capturing the temporal evolution of SOFA scores through GBTM may help identify distinct clinical courses that are not evident from initial assessments alone.

In addition, the early identification of such trajectory patterns at ICU admission remains challenging. Integrating machine learning (ML) methods with routinely available clinical data may offer a practical approach to support early trajectory stratification. Furthermore, interpretable ML techniques such as SHapley Additive exPlanations (SHAP) can facilitate clinical understanding by highlighting key baseline features associated with different disease courses.

Accordingly, the objectives of this study were to (1) identify distinct SOFA score trajectory groups in ICU patients with SA-BSI using GBTM, and (2) develop and externally validate an interpretable ML model to predict trajectory group membership using only ICU admission data. Through this two-pronged approach, we aimed to deepen understanding of the dynamic progression of OD in SA-BSI and explore its clinical implications for early phenotyping and individualized ICU management.

## Methods

### Study design and data collection

This retrospective observational study included two independent cohorts of ICU patients with SA-BSI. The development cohort was derived from the Medical Information Mart for Intensive Care IV (MIMIC-IV, v3.0) database, a publicly accessible resource containing deidentified ICU data between 2008 and 2022 (Johnson et al., 2025; Goldberger et al., 2000). The external validation cohort comprised patients admitted to the ICUs of the Second Affiliated Hospital of Zhejiang University School of Medicine (SAHZU), Hangzhou, China, between January 2013 and June 2024. Ethical approvals were obtained from both institutions (see **Supplementary Material** for further details).

For the SAHZU cohort, patients were identified through positive blood cultures for *S. aureus*. Only the first SA-BSI episode per ICU admission was retained. Exclusion criteria included age <18 years, hospital or survival time <24 hours, incomplete data, and classification as nonpathogenic *S. aureus* isolates (Zheng et al., 2020) (also see **Supplementary Material**). The same criteria were applied to the MIMIC cohort, except for isolate pathogenicity, which could not be determined from the database.

Demographic characteristics, comorbidities, laboratory variables, and clinical outcomes were extracted from institutional electronic medical record systems (EMRS). Comorbidities were identified via the International Classification of Diseases (ICD) -9 and ICD-10 codes (**Supplementary Table S1**). The primary outcomes were in-hospital and 28-day mortality; secondary outcomes included 90-day mortality and survival time. Additional details regarding data access, extraction procedures, and institutional approvals are provided in **Supplementary Methods**.

### Definitions and SOFA score collection

SA-BSI was diagnosed according to the Centers for Disease Control and Prevention definitions for BSI events (4psc_clabscurrent.pdf, 2025). BSI onset (Day 0) was designated as the date when the first culture-positive blood sample was collected. Nosocomial BSI was defined as a first positive culture obtained ≥48 hours after hospital admission in patients with no evidence of infection on admission (Garner et al., 1988; Billington et al., 2014). Sepsis and septic shock were diagnosed using the Sepsis-3 criteria (Singer et al., 2016).

OD was evaluated using the SOFA scores (Vincent et al., 1996), which were recorded daily from 1 day before to 3 days after BSI onset (Days -1 to +3). This time window was chosen to capture early OD dynamics during the critical diagnostic period prior to microbiological confirmation, and is consistent with prior studies demonstrating peak inflammatory responses during this interval (Gu et al., 2024), and the typical 3–5-day period required for blood culture results.

Because of the retrospective nature of the data and occasional absence of laboratory or physiologic values, not all patients had complete daily SOFA scores across the five-day period. However, the GBTM method applied in this study accommodates such longitudinal missingness through maximum likelihood estimation (MLE) (Bhavani et al., 2022); therefore, no imputation was performed for SOFA data.

### Group-based trajectory modeling

GBTM, a finite mixture modeling technique for identifying distinct longitudinal patterns within heterogeneous populations (Nagin and Odgers, 2010), was used to identify distinct temporal patterns of OD based on daily total SOFA scores. Modeling was performed on the MIMIC cohort and externally validated in the SAHZU cohort. Five time points were used for trajectory estimation: from 1 day before to 3 days after the onset of BSI (Days -1 to +3).

Due to the retrospective nature of the study and variable timing of laboratory and physiological measurements, not all patients had complete daily SOFA scores across the entire observation window. GBTM accommodates longitudinal missingness through a maximum likelihood estimation framework under the assumption that data are missing at random. Therefore, no imputation or forward-filling of SOFA scores or subscores was performed, and all available observations were used for trajectory estimation.

Candidate models with one to five trajectory groups and varying polynomial shapes (linear to cubic) were evaluated. Model selection was based on a combination of statistical and clinical criteria, including Bayesian Information Criterion (BIC), Akaike Information Criterion (AIC), entropy, posterior probabilities, and subgroup proportions, with the goal of balancing model fit, classification certainty, parsimony, and clinical interpretability (see **Supplementary Material** for detailed selection metrics and modeling criteria).

Each patient was assigned to a trajectory group based on the maximum posterior probability of membership. Classification reliability was further evaluated using average posterior probability metrics, with values ≥0.70 considered acceptable, as detailed in the **Supplementary Material**. External validation was conducted by applying the MIMIC-derived model parameters to the SAHZU cohort.

### Machine Learning Prediction and Model Interpretation of Trajectory Group

To evaluate whether ICU admission characteristics could predict subsequent OD trajectories, we developed a supervised ML model using Extreme Gradient Boosting (XGBoost). The primary outcome was trajectory group membership, which was defined by GBTM based on longitudinal SOFA scores.

Baseline features included demographic variables, vital signs, and laboratory results from the first 24 hours. To ensure compatibility across cohorts, only variables consistently available and harmonized using uniform clinical definitions and measurement units in both the MIMIC and SAHZU datasets were included. Missing values were imputed using median imputation.

Model training and internal validation were performed on the MIMIC cohort, which was randomly split into a training set (80%) and a validation set (20%). Given the imbalanced distribution of trajectory groups, model performance was evaluated on a per-class basis to ensure robust discrimination across all trajectory categories, including the smallest high-risk group. External validation was conducted using the SAHZU cohort by directly applying the trained MIMIC model without retraining or reparameterization. Recalibration was not performed because the primary objective of external validation was to assess discrimination and transportability of trajectory classification rather than to estimate absolute risk probabilities in the validation cohort. This approach allowed assessment of the model’s generalizability to an independent ICU population.

To interpret model predictions and identify key predictors of trajectory group assignment, SHAP values were calculated. SHAP summary plots were used to visualize both the relative importance of features and their directional influence on classification outcomes.

### Statistical analysis

To assess the association between SOFA trajectory groups and clinical outcomes, Cox proportional hazards models were fitted with in-hospital mortality as the primary endpoint. Covariates were selected based on clinical relevance and statistical significance (*p* < 0.05) in univariable analyses in either cohort (**Table 1**). Multivariable models included variables that remained significant after adjustment. Trajectory Group 1, representing the most stable clinical course, was used as the reference category.

**Table 1:** Demographics, comorbidities and other clinical characteristics for each trajectory group of the two cohorts.

Factor	Training cohort (MIMIC-IV)					Validation cohort (SAHZU)				
	Total (n = 834)	Group 1 (n = 402)	Group 2 (n = 334)	Group 3 (n = 98)	*P*	Total (n = 151)	Group 1 (n = 75)	Group 2 (n = 53)	Group 3 (n = 23)	*P*
Age, IQR	65.50 (53.00, 77.00)	64.50 (51.00,75.00)	67.00 (57.00,79.00)	60.50 (48.25,71.00)	**<.001**	57.00 (47.00, 70.50)	56.00 (45.00,65.00)	62.00 (53.00,75.00)	54.00 (41.50,74.00)	**.048**
Male, n(%)	503 (60.31)	214 (53.23)	217 (64.97)	72 (73.47)	**<.001**	115 (76.16)	54 (72.00)	45 (84.91)	16 (69.57)	.174
Race, n(%)					.205					–
ASIAN	18 (2.16)	7 (1.74)	9 (2.69)	2 (2.04)		151(100)	75(49.67)	53(35.10)	23(15.23)	
BLACK	97 (11.63)	40 (9.95)	44 (13.17)	13 (13.27)		0(0)	0(0)	0(0)	0(0)	
WHITE	132 (15.83)	60 (14.93)	49 (14.67)	23 (23.47)		0(0)	0(0)	0(0)	0(0)	
OTHER	587 (70.38)	295 (73.38)	232 (69.46)	60 (61.22)		0(0)	0(0)	0(0)	0(0)	
BMI, IQR	28.16 (24.53, 32.75)	27.97 (24.54,32.08)	27.94 (24.28,32.91)	30.19 (25.44,34.07)	.075	23.59 (20.79, 26.96)	23.88 (21.32,26.96)	23.07 (20.68,25.36)	23.18 (19.02,28.35)	.39
MRSA, n(%)	180 (21.58)	78 (19.40)	80 (23.95)	22 (22.45)	.32	118 (78.15)	59 (78.67)	39 (73.58)	20 (86.96)	.427
Nosocomial infection, n(%)	187 (22.42)	76 (18.91)	95 (28.44)	16 (16.33)	**.003**	100 (66.23)	47 (62.67)	37 (69.81)	16 (69.57)	.656
SOFA, IQR	4.00 (2.00, 7.00)	2.00 (1.00,3.75)	6.00 (4.00,7.00)	11.00 (9.00,13.00)	**<.001**	5.00 (3.00, 7.50)	3.00 (2.00,4.00)	7.00 (6.00,8.00)	11.00 (10.00,13.50)	**<.001**
Comorbidities										
Hypertension, n(%)	275 (32.97)	176 (43.78)	75 (22.46)	24 (24.49)	**<.001**	55 (36.42)	24 (32.00)	21 (39.62)	10 (43.48)	.506
Diabetes, n(%)	315 (37.77)	140 (34.83)	140 (41.92)	35 (35.71)	.129	23 (15.23)	10 (13.33)	10 (18.87)	3 (13.04)	.658
Chronic pulmonary disease, n(%)	203 (24.34)	96 (23.88)	83 (24.85)	24 (24.49)	.954	5 (3.31)	1 (1.33)	2 (3.77)	2 (8.70)	.186
Circulation diseases, n(%)	391 (46.88)	155 (38.56)	192 (57.49)	44 (44.90)	**<.001**	16 (10.60)	6 (8.00)	8 (15.09)	2 (8.70)	.416
Cerebrovascular disease, n(%)	143 (17.15)	54 (13.43)	67 (20.06)	22 (22.45)	**.02**	38 (25.17)	19 (25.33)	14 (26.42)	5 (21.74)	.91
Renal disease, n(%)	272 (32.61)	78 (19.40)	161 (48.20)	33 (33.67)	**<.001**	2 (1.32)	0 (0.00)	1 (1.89)	1 (4.35)	.13
Liver disease, n(%)	171 (20.50)	55 (13.68)	78 (23.35)	38 (38.78)	**<.001**	7 (4.64)	0 (0.00)	4 (7.55)	3 (13.04)	**.004**
Malignant cancer, n(%)	85 (10.19)	37 (9.20)	40 (11.98)	8 (8.16)	.362	8 (5.30)	4 (5.33)	3 (5.66)	1 (4.35)	1
Risk factors										
Invasive ventilation, n(%)	98 (11.75)	33 (8.21)	41 (12.28)	24 (24.49)	**<.001**	122 (80.79)	55 (73.33)	45 (84.91)	22 (95.65)	**.038**
Invasive procedure, n(%)	386 (46.28)	146 (36.32)	162 (48.50)	78 (79.59)	**<.001**	36 (23.84)	20 (26.67)	12 (22.64)	4 (17.39)	.638
RRT, n(%)	13 (1.56)	0 (0.00)	6 (1.80)	7 (7.14)	**<.001**	9 (5.96)	2 (2.67)	3 (5.66)	4 (17.39)	.056
CVC/PICC, n(%)	29 (3.48)	14 (3.48)	8 (2.40)	7 (7.14)	.079	121 (80.13)	54 (72.00)	47 (88.68)	20 (86.96)	**.045**
Treatments after BSI onset										
Invasive ventilation, n(%)	363 (43.53)	121 (30.10)	157 (47.01)	85 (86.73)	**<.001**	137 (90.73)	62 (82.67)	52 (98.11)	23 (100.00)	**.004**
RRT, n(%)	125 (14.99)	10 (2.49)	72 (21.56)	43 (43.88)	**<.001**	28 (18.54)	2 (2.67)	13 (24.53)	13 (56.52)	**<.001**
Outcomes										
Sepsis, n(%)	680 (81.53)	286 (71.14)	298 (89.22)	96 (97.96)	**<.001**	144 (95.36)	69 (92.00)	52 (98.11)	23 (100.00)	.212
Septic shock, n(%)	296 (35.49)	51 (12.69)	166 (49.70)	79 (80.61)	**<.001**	70 (46.36)	15 (20.00)	32 (60.38)	23 (100.00)	**<.001**
In-hospital mortality, n(%)	218 (26.14)	55 (13.68)	100 (29.94)	63 (64.29)	**<.001**	52 (34.44)	10 (13.33)	23 (43.40)	19 (82.61)	**<.001**
In-hospital LOS, IQR	13.81 (8.36, 23.83)	12.99 (8.85,21.51)	16.14 (8.61,27.07)	10.75 (3.63,21.97)	**<.001**	31.00 (16.50, 61.00)	36.00 (21.50,71.50)	27.00 (18.00,55.00)	21.00 (7.00,45.50)	**.041**
ICU LOS, IQR	3.60 (1.83, 7.40)	2.71 (1.62,5.57)	4.13 (2.10,8.75)	6.43 (2.81,11.16)	**<.001**	19.00 (9.00, 43.00)	19.00 (9.50,51.50)	22.00 (11.00,43.00)	10.00 (3.00,24.50)	.106
Survival time, IQR	374.00 (18.79, 530.75)	385.00 (86.46,643.00)	250.28 (13.94,468.75)	9.96 (3.17,384.00)	**<.001**	22.00 (9.00, 61.50)	37.00 (15.50,93.50)	19.00 (9.00,42.00)	5.00 (2.50,11.00)	**<.001**
Vital sign										
Temperature, °C, IQR	36.61 (36.28, 38.78)	36.72 (36.39,38.78)	36.56 (36.28,38.66)	36.44 (35.62,39.21)	**.014**	38.40 (37.50, 39.00)	38.40 (37.60,39.00)	38.40 (37.50,39.00)	38.50 (37.40,39.30)	.93
Heart rate, IQR	86.00 (65.00, 127.00)	84.00 (65.00,125.75)	85.00 (64.00,126.75)	118.50 (64.00,135.00)	.188	108.00 (89.50, 128.00)	103.00 (90.00,126.00)	107.00 (87.00,125.00)	126.00 (93.50,137.50)	.121
Respiratory rate, IQR	28.00 (11.00, 35.00)	27.00 (11.00,35.00)	29.00 (11.00,35.00)	30.25 (11.75,37.75)	.115	21.00 (18.00, 27.00)	20.00 (18.00,26.50)	22.00 (18.00,26.00)	20.00 (16.00,27.50)	.913
MAP, mmHg, IQR	58.00 (49.00, 66.00)	63.00 (53.00,75.00)	55.50 (47.00,62.00)	51.00 (43.00,58.00)	**<.001**	83.00 (71.83, 98.33)	89.67 (76.67,100.00)	76.00 (71.33,96.67)	75.33 (69.17,87.17)	**.008**
GCS, IQR	14.00 (13.00, 15.00)	15.00 (14.00,15.00)	14.00 (12.00,15.00)	14.00 (9.00,15.00)	**<.001**	15.00 (10.00, 15.00)	15.00 (12.00,15.00)	12.00 (7.00,15.00)	12.00 (9.00,15.00)	**<.001**
Blood routine test										
WBC, ×10^9/L, IQR	15.30 (10.70, 21.60)	14.70 (10.75,20.00)	15.60 (10.38,22.33)	17.60 (11.88,25.30)	**.028**	11.00 (8.35, 16.35)	10.60 (8.30,15.25)	12.50 (8.30,17.20)	11.00 (8.45,17.25)	.68
ANC, ×10^9/L, IQR	12.23 (8.58, 17.73)	12.15 (8.79,17.55)	12.06 (8.09,18.17)	13.30 (8.86,18.52)	.777	9.35 (6.65, 14.62)	8.34 (6.72,12.97)	10.85 (6.48,16.11)	9.35 (7.24,15.93)	.442
Hemoglobin, g/L, IQR	69.00 (62.00, 82.00)	71.00 (64.00,84.75)	66.00 (61.00,78.00)	68.50 (62.00,81.00)	**<.001**	95.00 (78.50, 110.50)	99.00 (83.50,110.50)	91.00 (79.00,108.00)	87.00 (74.00,119.50)	.598
Hematocrit, %, IQR	29.30 (25.42, 33.50)	29.40 (25.90,33.25)	29.30 (25.00,33.73)	28.85 (24.92,33.27)	.535	28.40 (24.05, 33.05)	29.20 (24.80,32.65)	28.10 (24.00,33.10)	26.20 (21.95,35.25)	.54
Chemistry										
ALT, U/L, IQR	28.00 (17.00, 52.00)	23.00 (15.00,41.00)	28.00 (17.00,53.00)	41.00 (24.50,139.00)	**<.001**	41.00 (27.25, 77.00)	39.00 (25.50,71.50)	44.00 (30.50,77.25)	41.00 (27.00,71.00)	.605
AST, U/L, IQR	45.00 (26.00, 79.00)	33.00 (21.25,58.00)	49.00 (28.00,82.00)	84.00 (48.00,270.50)	**<.001**	45.50 (30.25, 70.75)	38.00 (28.00,62.50)	49.00 (33.00,71.25)	58.00 (36.00,91.00)	**.038**
TBil, μmol/L, IQR	15.39 (8.55, 30.78)	11.97 (7.27,18.81)	17.10 (10.26,40.19)	32.49 (17.10,69.68)	**<.001**	17.80 (11.30, 28.48)	13.10 (9.30,19.60)	19.00 (14.50,30.80)	34.10 (22.35,45.25)	**<.001**
BUN, IQR	32.00 (19.00, 56.00)	21.00 (14.50,34.50)	42.00 (28.00,62.00)	58.00 (41.00,72.75)	**<.001**	9.20 (5.88, 13.50)	6.94 (4.90,9.62)	12.49 (6.88,17.31)	13.05 (10.13,18.57)	**<.001**
SCr, μmol/L, IQR	123.76 (79.56, 247.52)	88.40 (61.88,132.60)	176.80 (106.08,335.92)	274.04 (167.96,499.46)	**<.001**	68.00 (47.90, 102.90)	53.00 (41.00,70.00)	92.00 (61.00,129.00)	115.00 (72.45,252.00)	**<.001**
Coagulation										
INR, IQR	1.40 (1.20, 1.90)	1.30 (1.20,1.60)	1.50 (1.30,2.00)	1.80 (1.40,2.50)	**<.001**	1.21 (1.12, 1.38)	1.17 (1.07,1.32)	1.21 (1.14,1.42)	1.36 (1.21,1.69)	**.012**
PTT, IQR	33.40 (29.00, 42.90)	31.60 (28.20,36.80)	33.90 (29.70,45.65)	41.90 (34.50,52.90)	**<.001**	40.15 (35.97, 46.18)	39.60 (35.50,44.20)	39.50 (36.90,45.50)	43.80 (39.73,52.65)	.058

Biological indicators including vital signs, laboratory findings, and SOFA score are measured at the onset day of SA-BSI. IQR, interquartile range; BMI, body mass index; RRT, renal replacement therapy; CVC, central venous catheter; PICC, peripherally inserted central venous catheters; MRSA, Methicillin-resistant *Staphylococcus aureus*; BSI, bloodstream infection; LOS, length of stay; MAP, mean arterial pressure; GCS, Glasgow coma scale; WBC, white blood cells; ANC, absolute neutrophil count; ALT, alanine transaminase; AST, aspartate transferase; TBil, total bilirubin; BUN, blood urea nitrogen; SCr, serum creatinine; INR, international normalized ratio; PTT, partial thromboplastin time; SOFA, sequential organ failure assessment. The bold values indicate statistically significant P values (P < 0.05).

Kaplan-Meier survival curves were used to compare 28-day and 90-day mortality across trajectory groups, with statistical differences assessed by the log-rank test. All statistical analyses were performed using R, Python, and Stata software. Additional details are provided in the **Supplementary Material**.

## Results

### Patient admission

A total of 834 ICU patients from the MIMIC-IV cohort and 151 from the SAHZU cohort were included in the final analysis (**Supplementary Figure S1**). In the MIMIC-IV cohort, 2,171 patients with SA-BSI were initially identified. After excluding 1,260 non-ICU admissions, 25 with hospital LOS <24 hours, 3 with survival <24 hours, and 49 with incomplete data, 834 patients remained.

In the SAHZU cohort, 641 SA-BSI cases were identified. After excluding 434 non-ICU admissions, 5 with LOS <24 hours, 6 with survival <24 hours, 22 with incomplete data, and 17 with nonpathogenic *S. aureus* isolates, 151 ICU patients were included in the final cohort.

### GBTM modeling results

To identify distinct patterns of OD, we applied GBTM to daily SOFA scores from Days -1 to +3. In the MIMIC cohort, models with one to five trajectory groups were tested using various polynomial forms. As shown in **Supplementary Table S2**, model fit progressively improved with more groups, reflected by increasing log-likelihood and decreasing AIC/BIC values. However, the gain in entropy plateaued between the two-group (0.833) and three-group (0.829) models, and declined further thereafter.

The five-group model included a subgroup comprising only 3.6% of patients, while the four-group model had a group size of 6.6%, both raising concerns about overfitting and reduced classification certainty. In contrast, the three-group model balanced improved fit with robust entropy and clinically meaningful distribution (48.2%, 39.9%, and 11.9%). Based on statistical robustness, group size stability, and clinical interpretability, the three-group solution was selected. Full model selection criteria are presented in the **Supplementary Material**.

**Figure 1:**
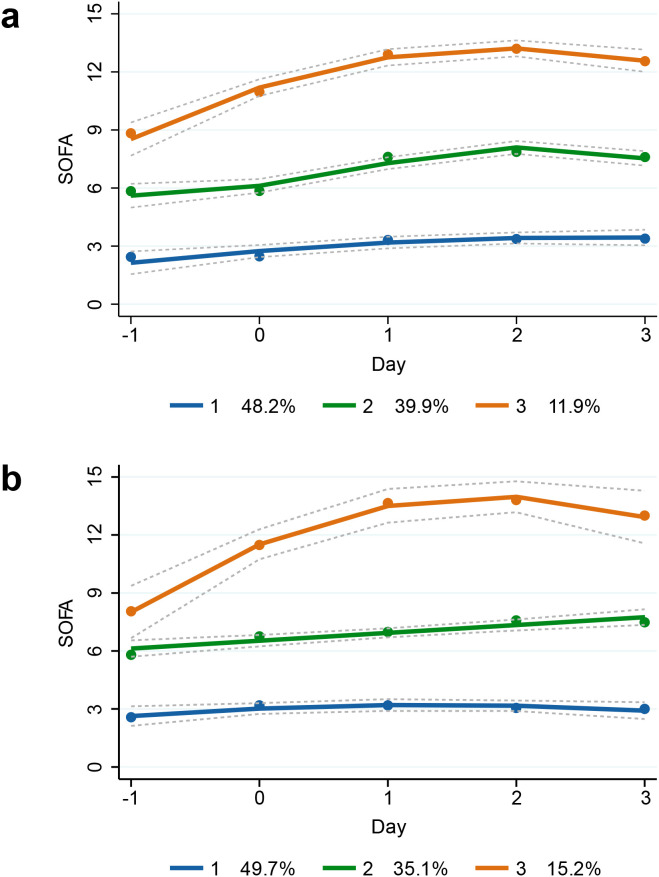
GBTM of SOFA scores in ICU patients with SA-BSI. Trajectory groups were identified using GBTM based on daily SOFA scores from Day -1 to Day +3 relative to the onset of SA-BSI in **(A)** the MIMIC-IV cohort and **(B)** the SAHZU cohort. Three distinct and reproducible trajectories were observed in both cohorts, representing Group 1: stable, Group 2: moderately worsening, and Group 3: severely deteriorating OD patterns. Solid lines depict estimated mean SOFA trajectories, and dashed lines denote 95% confidence intervals. The proportions of patients assigned to each trajectory group are displayed in the legends for both cohorts, demonstrating comparable distribution patterns and supporting the external generalizability of the trajectory structure. GBTM, group-based trajectory modeling; SOFA, Sequential Organ Failure Assessment; ICU, intensive care unit; SA-BSI, *Staphylococcus aureus* bloodstream infection; MIMIC-IV, Medical Information Mart for Intensive Care IV; SAHZU, Second Affiliated Hospital of Zhejiang University; OD, organ dysfunction.

**Figures 1A, B** display the SOFA score trajectories for the three identified groups in the MIMIC-IV and SAHZU cohorts, respectively. Group 1 (48.2% in MIMIC, 49.7% in SAHZU) exhibited persistently low SOFA scores, suggesting a stable clinical course. Group 2 (39.9% in MIMIC, 35.1% in SAHZU) showed moderate SOFA scores with a mild upward trend. Group 3 (11.9% in MIMIC, 15.2% in SAHZU) had the highest initial SOFA scores, peaking on Day 2 and then declining slightly. Trajectories of SOFA subscores for each group are presented in the **Supplementary Figure S2**.

### Baseline characteristics

Baseline characteristics of ICU patients with SA-BSI across the three SOFA trajectory groups are summarized in **Table 1**. Notably, in both cohorts, patients in Group 3 were the youngest, while those in Group 2 tended to be the oldest.

Groups 2 and 3 had significantly higher proportions of patients requiring invasive mechanical ventilation and renal replacement therapy (RRT). Clinical outcomes progressively worsened across the groups: Group 3 exhibited the highest incidence of septic shock (80.61% in MIMIC; 100% in SAHZU), the highest in-hospital mortality (64.29% in MIMIC; 82.61% in SAHZU), and the shortest survival durations.

Biological markers and SOFA subscores at SA-BSI onset also demonstrated consistent gradients. Initial SOFA scores were lowest in Group 1 and highest in Group 3 across both cohorts. OD markers, including total bilirubin (TBil), blood urea nitrogen (BUN), and serum creatinine (SCr), increased progressively from Group 1 to Group 3. Coagulation parameters such as international normalized ratio (INR) and partial thromboplastin time (PTT) were also most abnormal in Group 3.

Additional laboratory indicators available only in the SAHZU cohort are presented in **Supplementary Table S3**. Inflammatory markers (e.g., CRP, procalcitonin [PCT]), coagulation indicators (e.g., D-dimer), and metabolic stress markers (e.g., lactate) were markedly elevated in Group 3, reflecting a severe inflammatory and hypercoagulable state. Arterial blood gas results also revealed more pronounced respiratory compromise in this group.

### SOFA subscore trajectories across groups and cohorts

**Figure 2:**
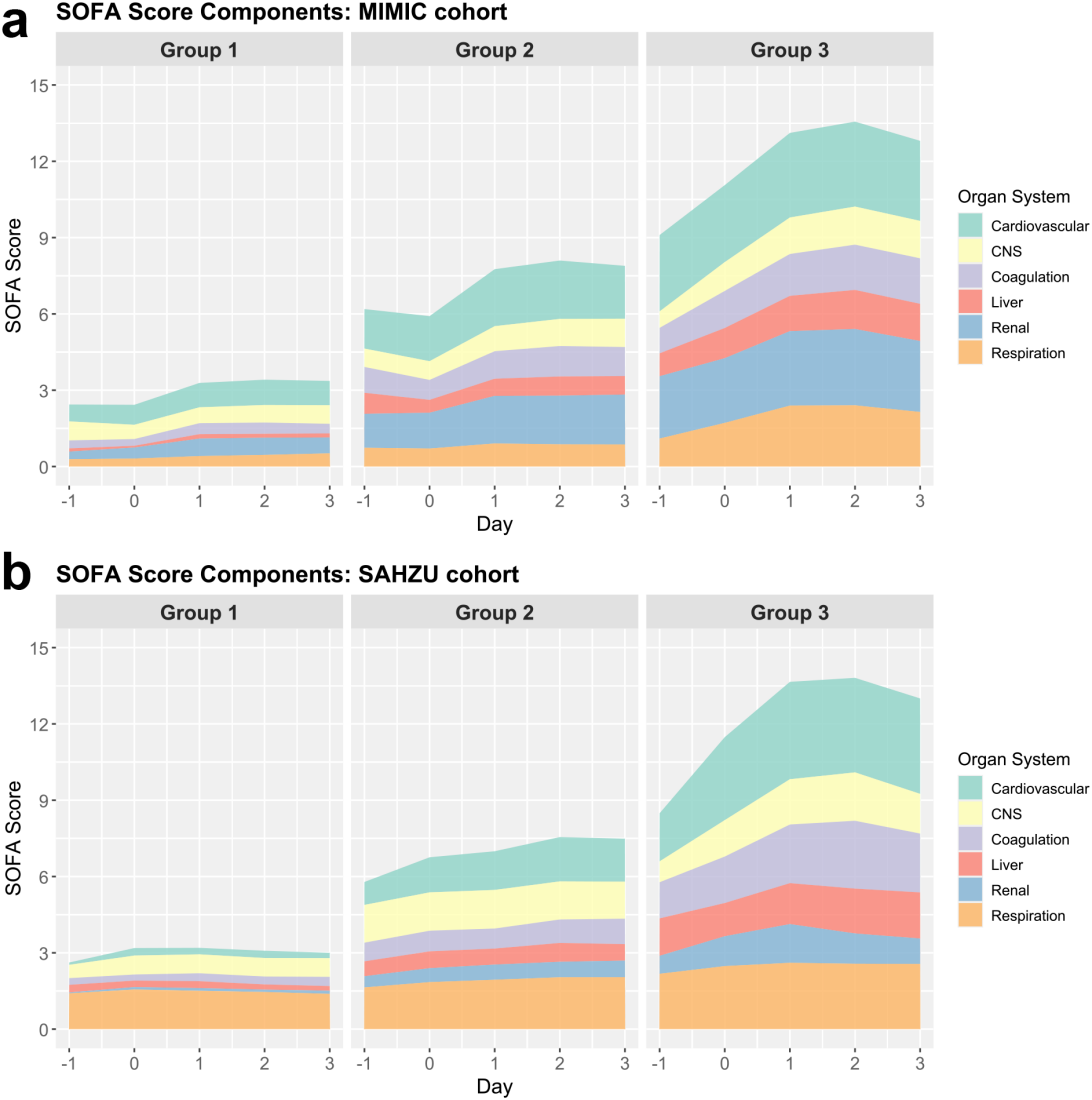
Temporal evolution of SOFA score components across trajectory groups in ICU patients with SA-BSI. Stacked area plots depict the contributions of individual SOFA subcomponents from Day -1 to Day +3 within each trajectory group in **(A)** the MIMIC cohort and **(B)** the SAHZU cohort. Group 1 demonstrated minimal fluctuations across all organ systems, consistent with stable organ function. Group 2 showed progressive increases primarily in cardiovascular and renal components. Group 3 exhibited early and pronounced increases across multiple organ systems, particularly the cardiovascular, renal, and respiratory domains, reflecting rapidly worsening multi-organ dysfunction. SOFA, Sequential Organ Failure Assessment; ICU, intensive care unit; SA-BSI, *Staphylococcus aureus* bloodstream infection; MIMIC, Medical Information Mart for Intensive Care; SAHZU, Second Affiliated Hospital of Zhejiang University; CNS, central nervous system.

The temporal dynamics of individual SOFA subscores varied distinctly across trajectory groups and between cohorts, as illustrated in **Figure 2** (stacked area charts) and **Supplementary Figure S2** (line plots). In the MIMIC cohort, Group 1 maintained low and stable subscores throughout the observation window, reflecting minimal and non-progressive OD. Group 2 exhibited a modest upward trend, primarily driven by progressive increases in cardiovascular and renal scores. Group 3 showed early and sharp elevations across multiple subscores, peaking around Day 2, with cardiovascular dysfunction being the predominant contributor, followed by renal and respiratory involvement.

In the SAHZU cohort, Group 1 showed a similar low-risk profile to MIMIC. However, respiratory scores were persistently elevated across all groups, particularly in Group 2, suggesting a higher baseline burden of pulmonary dysfunction. In Group 3, cardiovascular and respiratory components showed pronounced increases, while renal dysfunction was comparatively less prominent than in MIMIC. These patterns suggest cohort-specific differences in OD, yet cardiovascular failure remained the central and most consistent driver of trajectory deterioration across both populations.

Taken together, these findings highlight the heterogeneity of MOD patterns across trajectory groups and reinforce the prognostic significance of cardiovascular compromise in patients with SA-BSI.

### Outcomes

Trajectory classification was strongly associated with in-hospital mortality (**Supplementary Table S4**). In multivariable Cox regression analysis (**Supplementary Table S5**), the trajectory group remained an independent predictor of in-hospital mortality after adjusting for key clinical covariates. In the MIMIC cohort, patients in Group 2 and Group 3 had progressively higher mortality risks compared to Group 1, with hazard ratios (HRs) of 1.56 (95% confidence interval [CI]: 1.27–1.91, *p* < 0.001) and 4.60 (95% CI: 3.49–6.07, *p* < 0.001), respectively. In the SAHZU cohort, Group 3 also showed significantly elevated mortality risk (HR: 5.36, 95% CI: 2.87–10.00, *p* < 0.001), confirming the robustness of the trajectory grouping across distinct populations.

**Figure 3:**
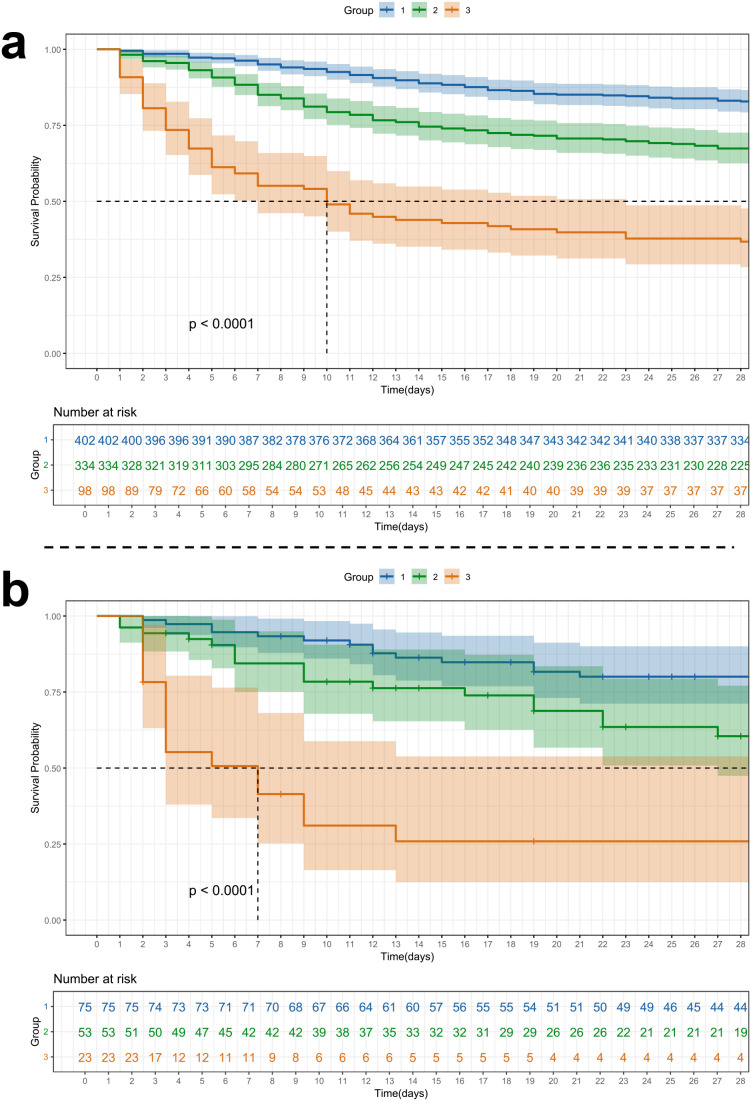
Kaplan-Meier curves showing 28-day survival probabilities across SOFA trajectory groups in patients with SA-BSI. Kaplan-Meier survival curves are stratified by trajectory group for **(A)** the MIMIC cohort and **(B)** the SAHZU cohort. Group 1 demonstrated the highest survival probability, followed by Group 2, whereas Group 3 showed markedly reduced survival. Shaded areas indicate 95% confidence intervals. Survival differences across groups were statistically significant in both cohorts (log-rank test, *p* < 0.0001). The number of patients at risk at each time point is presented beneath the plots. SOFA, Sequential Organ Failure Assessment; SA-BSI, *Staphylococcus aureus* bloodstream infection; MIMIC, Medical Information Mart for Intensive Care; SAHZU, Second Affiliated Hospital of Zhejiang University.

Kaplan-Meier survival curves further supported these findings (**Figure 3**; **Supplementary Figure S3**), revealing significant differences in 28-day and 90-day survival across trajectory groups (log-rank test, *p* < 0.001). Patients in Group 1 had the highest survival probabilities, while those in Group 3 experienced the most rapid decline. Interestingly, the survival difference between Groups 1 and 2 reached statistical significance in the MIMIC cohort but not in SAHZU (**Supplementary Table S6**), suggesting possible cohort-specific effects or sample size limitations in the external validation set.

### Machine learning prediction and interpretation of trajectory groups

The XGBoost model accurately classified patients into the three predefined SOFA trajectory groups using only baseline ICU admission features. In internal validation within the MIMIC cohort, the model demonstrated excellent discrimination across all trajectory classes, with the Area Under the Receiver Operating Characteristic Curve (AUC) values of 0.904, 0.855, and 0.966 for Groups 1, 2, and 3, respectively (**Table 2**). Sensitivity ranged from 0.667 to 0.846, and specificity from 0.775 to 0.979, indicating balanced performance across stable and deteriorating patterns. External validation using the SAHZU cohort yielded similarly robust results, with AUCs of 0.964, 0.899, and 0.920 for the three groups, respectively, supporting the model’s generalizability across distinct ICU populations.

**Table 2:** Performance of the XGBoost model in predicting SOFA trajectory groups in ICU SA-BSI patients.

Metrics	Internal test set			External validation set		
	Group 1	Group 2	Group 3	Group 1	Group 2	Group 3
Sensitivity	0.846	0.667	0.783	0.960	0.642	0.609
Specificity	0.775	0.842	0.979	0.750	0.898	0.984
PPV	0.767	0.733	0.857	0.791	0.773	0.875
NPV	0.852	0.794	0.966	0.950	0.822	0.933
Accuracy	0.808	0.772	0.952	0.854	0.808	0.927
AUC	0.904	0.855	0.966	0.964	0.899	0.920

Summary of classification metrics for each SOFA trajectory group, derived from the XGBoost model using early ICU admission data. Metrics are reported separately for the internal test set (MIMIC-IV cohort) and external validation set (SAHZU cohort). Values include sensitivity, specificity, PPV, NPV, accuracy, and AUC, reflecting model performance in predicting Group 1 (stable), Group 2 (moderately worsening), and Group 3 (severely deteriorating) trajectories. SOFA, Sequential Organ Failure Assessment; ICU, Intensive Care Unit; SA-BSI, *Staphylococcus aureus* bloodstream infection; PPV, Positive Predictive Value; NPV, Negative Predictive Value; AUC, Area Under the Receiver Operating Characteristic Curve; MIMIC-IV, Medical Information Mart for Intensive Care IV; SAHZU, Second Affiliated Hospital of Zhejiang University.

**Figure 4:**
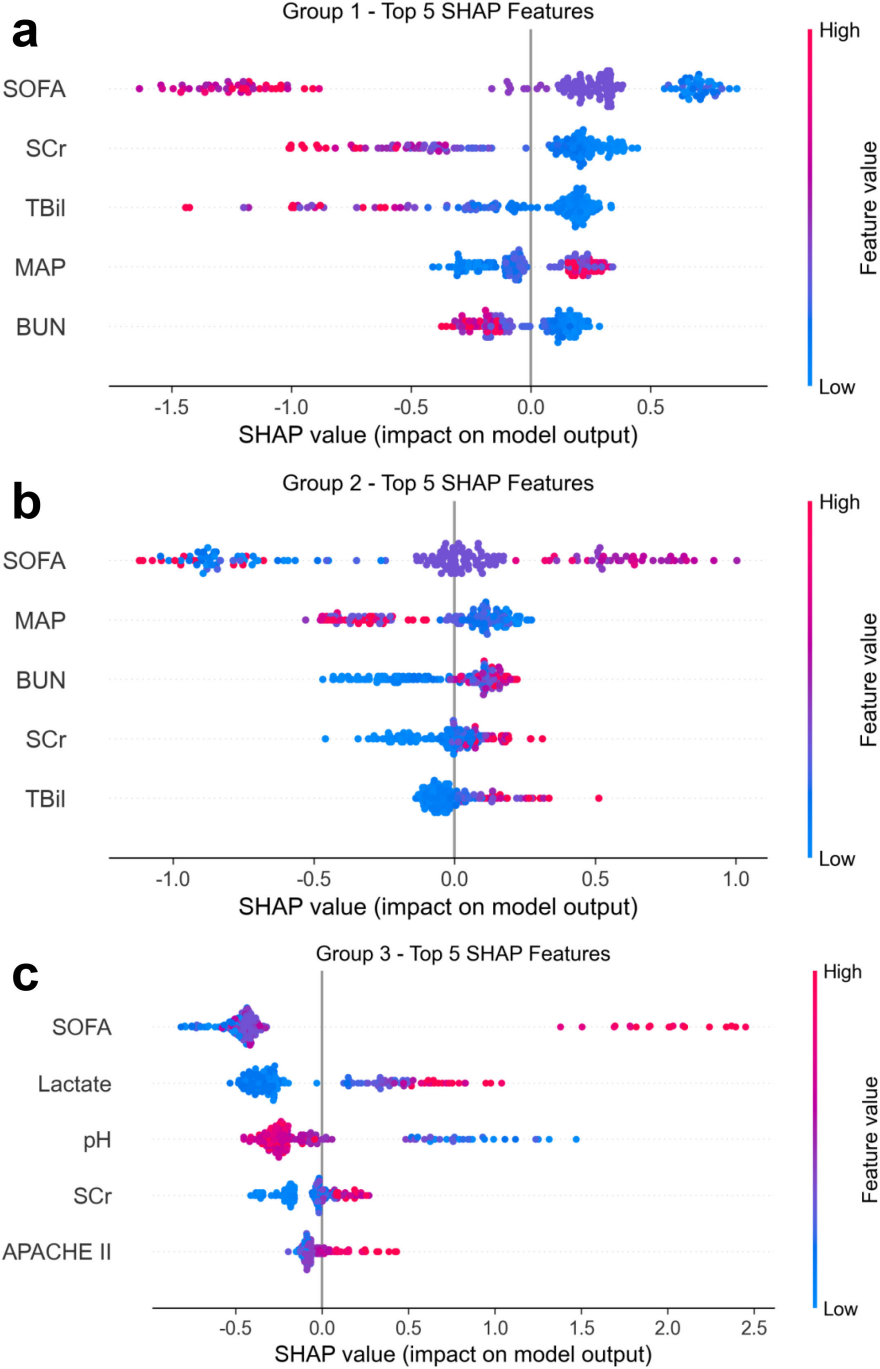
SHAP analysis of feature contributions to predicted SOFA trajectory group membership. SHAP summary plots illustrate the top five baseline features contributing to the XGBoost model’s classification of patients into **(A)** Group 1 (stable), **(B)** Group 2 (moderately worsening), and **(C)** Group 3 (severely deteriorating) SOFA trajectory groups. Each point represents an individual patient, with color indicating the corresponding feature value (blue = low, red = high). SHAP values on the x-axis quantify the direction and magnitude of each feature’s impact on model output. For Group 1, lower SOFA scores, serum creatinine (SCr), and total bilirubin (TBil) were associated with greater likelihood of assignment. Group 2 classification was driven primarily by mean arterial pressure (MAP), blood urea nitrogen (BUN), and renal-related indicators. Group 3 membership was most influenced by higher SOFA scores, elevated lactate, and lower pH, reflecting profound metabolic and hemodynamic derangements characteristic of severe OD. SHAP, SHapley Additive exPlanations; SOFA, Sequential Organ Failure Assessment; SCr, serum creatinine; TBil, total bilirubin; MAP, mean arterial pressure; BUN, blood urea nitrogen; OD, organ dysfunction.

SHAP-based interpretation of the trained model revealed distinct baseline predictors for each trajectory group (**Figure 4**). For Group 1, a higher probability of classification was associated with lower admission SOFA scores, SCr, and TBil, suggesting preserved organ function. In Group 2, mean arterial pressure (MAP) and renal function markers (BUN, SCr) were the most influential, reflecting early circulatory and renal compromise. Group 3 was primarily driven by elevated lactate, high admission SOFA and Acute Physiology and Chronic Health Evaluation (APACHE) II scores, and low arterial pH, pointing to metabolic acidosis and severe MOD as key determinants of a rapidly deteriorating clinical course.

These results indicate that early ICU data can reliably predict subsequent OD trajectories. Moreover, SHAP-derived feature contributions offer interpretable insights into the underlying pathophysiological patterns unique to each clinical phenotype.

## Discussion

This study identified several novel and clinically relevant findings. First, by analyzing longitudinal SOFA score trajectories over the early course of SA-BSI, we uncovered three distinct OD patterns that were consistently reproducible across two independent ICU cohorts and strongly associated with clinical outcomes. Higher-risk trajectory groups showed significantly increased mortality, underscoring the prognostic value of dynamic OD assessment. Second, biological markers and SOFA subscores demonstrated progressive deterioration from Group 1 to Group 3, with cardiovascular dysfunction emerging as the predominant contributor to adverse outcomes. Third, in the SAHZU cohort, the availability of inflammatory and coagulation biomarkers (e.g., CRP, PCT, D-dimer) provided additional insight into systemic responses not captured by SOFA alone, highlighting the potential of multidimensional risk profiling. Finally, we demonstrated that ML using early ICU data can accurately predict trajectory group membership. The incorporation of SHAP-based interpretability further revealed physiologically coherent predictors, such as preserved renal function for favorable courses and elevated lactate and acidosis for severe deterioration, supporting both the transparency and clinical relevance of this approach. Together, these findings suggest that combining trajectory modeling with interpretable ML offers a feasible and informative strategy for early risk stratification in SA-BSI.

While prior studies have demonstrated the prognostic value of short-term (e.g., 72-hour) SOFA score changes in sepsis populations (Minne et al., 2008; Xu et al., 2022), our findings extend this understanding by emphasizing the importance of sustained longitudinal trends. By capturing dynamic patterns of OD over a five-day window, our trajectory-based approach identified heterogeneous clinical courses that may be overlooked by static or early SOFA measurements. Rather than modeling the entire ICU stay, this focused early window was intentionally selected to reflect the clinically actionable phase surrounding SA-BSI onset, during which OD evolves rapidly and key management decisions are often required before definitive microbiological confirmation. This is consistent with previous research in other ICU populations (Zhou et al., 2020; Yu et al., 2024), including our own study in critically ill COVID-19 patients, where four distinct SOFA trajectories were identified and the most severe group exhibited markedly higher 28-day mortality (Yu et al., 2024). In SA-BSI, we observed a similar pattern: Group 3, defined by persistently elevated SOFA scores, was strongly predictive of adverse outcomes. An additional insight from our trajectory analysis was the divergent SOFA progression patterns between Groups 2 and 3. Group 2 exhibited a steady increase in SOFA scores, whereas Group 3 peaked early and then declined slightly while remaining critically elevated. Despite this apparent improvement, Group 3 had significantly worse outcomes, suggesting that short-term decreases in SOFA scores do not necessarily indicate recovery and may instead mask persistent OD or ongoing systemic inflammation—key drivers of mortality in SA-BSI (Minne et al., 2008; Zorowitz, 2016). These findings highlight the limitations of relying solely on static or short-interval SOFA changes for prognostication. It should also be noted that the intermediate trajectory group likely encompasses patients with heterogeneous clinical courses, including individuals who subsequently stabilize as well as those who progress toward more severe OD, which may partly explain its intermediate prognostic profile. Notably, these trajectories were not only descriptive but also predictable. Using an XGBoost classifier trained on ICU admission data, we accurately assigned patients to their eventual trajectory groups. SHAP interpretation further enhanced model utility by providing biologically coherent explanations. For example, preserved renal and hepatic function favoring stable courses, while elevated lactate and low pH predicted deterioration. By identifying high-risk phenotypes shortly after blood culture collection (before definitive microbiological results become available), trajectory-informed ML models may support timely triage, guide early intervention, and optimize empiric antimicrobial therapy during this critical diagnostic window. In this context, the SOFA trajectory observation window (Days −1 to +3) was designed to align with the early diagnostic phase of SA-BSI, when clinical decisions frequently need to be made in the absence of definitive microbiological confirmation.

Cardiovascular dysfunction emerged as the dominant contributor to poor outcomes, particularly in the highest-risk trajectory group. In Group 3, cardiovascular SOFA subscores remained persistently elevated, indicating sustained circulatory failure closely linked to increased mortality. This finding echoes prior evidence that cardiovascular compromise plays a central role in ICU sepsis mortality (Snell and Parrillo, 1991; Mayr et al., 2006), contributing to impaired perfusion, metabolic derangements, and systemic inflammation. In the context of SA-BSI, this pattern may be further driven by pathogen-specific mechanisms, including toxin-mediated endothelial injury, superantigen-induced hyperinflammatory responses, and sepsis-associated myocardial depression, all of which can exacerbate vasoplegia and circulatory collapse (Powers et al., 2012; Touaitia et al., 2025). Interestingly, this contrasts with our previous work in critically ill COVID-19 patients (Yu et al., 2024), where respiratory dysfunction was the predominant driver of poor prognosis. Such divergence highlights the disease-specific nature of OD evolution and underscores the need for tailored risk assessment strategies in different syndromes. By decomposing SOFA trajectories into organ-specific components, our approach provides granular insight into the pathophysiology of SA-BSI and may better guide resource allocation, such as early circulatory support, in patients at highest risk.

The availability of inflammatory and coagulation biomarkers in the SAHZU cohort offered valuable complementary insights into systemic responses underlying OD progression. In Group 3, markers such as CRP, PCT, and D-dimer were markedly elevated, consistent with a hyperinflammatory and hypercoagulable state that paralleled severe OD. These findings reinforce the well-established role of systemic inflammation and coagulopathy in driving poor outcomes in critical illness (Strauss et al., 2001; Brummel et al., 2024), and support a multidimensional risk stratification approach that integrates both physiological and biochemical markers. Notably, although both Groups 2 and 3 had elevated mortality in both cohorts, only the MIMIC cohort showed a statistically significant survival difference between Groups 1 and 2 (**Supplementary Table S6**). This discrepancy may reflect sample size limitations or unmeasured cohort-specific factors, but also suggests that the additional biomarker data available in SAHZU allowed finer risk discrimination, particularly among patients with similar SOFA trajectories. Together, these observations highlight the added value of combining trajectory-based modeling with dynamic biomarkers to more comprehensively capture the heterogeneity and severity of SA-BSI. Given that these inflammatory and coagulation biomarkers were available only in the SAHZU cohort, their associations with trajectory patterns were explored in a descriptive and hypothesis-generating manner, and should be interpreted cautiously pending validation in larger, multi-cohort studies.

Beyond trajectory characterization, we further examined the relationship between baseline severity and longitudinal risk patterns. In multivariable Cox regression analyses, baseline SOFA score was no longer independently associated with mortality after adjustment for trajectory group membership. This finding likely reflects the fact that trajectory classification captures longitudinal patterns of OD, integrating both baseline severity and subsequent disease evolution over time. Accordingly, trajectory group membership provides prognostic information beyond that of a single time-point SOFA measurement, rather than diminishing the clinical relevance of baseline SOFA itself. Building on this, our ML model further demonstrated that early ICU data could accurately predict patients’ subsequent OD trajectories. The XGBoost classifier, trained solely on features available within the first 24 hours of admission, achieved excellent discrimination across all trajectory groups, including in external validation. This underscores that early physiological and laboratory indicators already carry prognostic signals reflecting the likely clinical course in SA-BSI. Importantly, SHAP-based model interpretation enhanced clinical transparency by identifying trajectory-specific predictors. Preserved renal and hepatic function characterized stable courses, whereas elevated lactate, low arterial pH, and high APACHE II scores were strong predictors of deterioration. Unlike conventional risk models focused on static outcomes (e.g., mortality), this approach anticipates dynamic disease evolution, enabling earlier recognition of patients at risk for progressive OD. As such, interpretable ML offers a promising tool for real-time triage and personalized management during the diagnostic window when culture results remain pending.

This study focuses on the early-stage SOFA score trajectories in patients with SA-BSI, aiming to integrate trajectory-based assessment into clinical practice during the period when microbiological results are still pending. This approach offers several potential benefits. First, it provides a framework for continuous and dynamic evaluation of OD, enabling clinicians to capture evolving physiological changes rather than relying solely on static SOFA values. Second, trajectory group classification serves as a practical tool for early ICU risk stratification. During this early clinical period, trajectory-based information should be interpreted as a dynamic indicator of evolving risk rather than as a definitive prognostic endpoint. Trajectories trending toward higher-risk patterns may signal the need for closer monitoring and heightened clinical vigilance before microbiological confirmation, whereas stable trajectories may suggest a lower immediate risk of rapid deterioration. By identifying patients most likely to deteriorate, this method may help optimize resource allocation and guide the timing and intensity of empiric antimicrobial therapy, particularly during the diagnostic window before culture confirmation. For instance, patients classified in Group 3 may benefit from early escalation of cardiovascular support and broad-spectrum antibiotics, whereas Group 2 patients may require closer hemodynamic monitoring and tailored interventions to prevent further deterioration. In contrast, Group 1 patients with stable OD may be managed less intensively, thereby facilitating efficient ICU triage. Third, trajectory-based modeling holds promise for improving clinical trial design. Stratifying patients by trajectory-defined risk can enhance cohort homogeneity, enabling interventions to be evaluated within more phenotypically consistent subgroups and thereby improving both statistical power and translational relevance in infectious disease trials. Finally, integrating automated trajectory calculations into electronic health records (EHRs) could enable real-time decision support for clinicians. By flagging patients who begin to follow high-risk trajectories, such systems may prompt timely interventions and more personalized treatment strategies, including targeted antimicrobial stewardship. Collectively, these applications align with the broader goals of dynamic infection surveillance and precision critical care in the ICU. Importantly, the trajectory-based framework is intended to support early risk stratification and clinical decision-making, rather than to replace clinician judgment or to serve as direct guidance for specific therapeutic interventions. Focusing specifically on SA-BSI provides additional insight beyond sepsis in general, as SA-BSI represents a distinct clinical entity characterized by unique virulence factors, a propensity for persistent bacteremia, and a high burden of cardiovascular complications. By examining SOFA trajectories within this pathogen-specific context, our study highlights patterns of OD evolution that may be diluted or obscured in more heterogeneous sepsis populations.

This study has several limitations. First, as a retrospective analysis, selection bias and missing data inevitably influenced our findings, particularly in the MIMIC-IV cohort, where detailed microbiological and laboratory information, such as inflammatory markers, was unavailable. These gaps reduced the granularity of trajectory characterization and limited direct comparability between cohorts. To mitigate missingness, we leveraged the inherent strengths of GBTM in handling incomplete longitudinal data via MLE and provided detailed data availability summaries (**Supplementary Table S7**) to enhance transparency and reproducibility. Second, differences between the MIMIC and SAHZU cohorts in demographics, comorbidities, ICU admission thresholds, clinical management, and biomarker availability, as well as differences in laboratory measurement frequency and data collection practices, may have contributed to variations in trajectory distribution and survival patterns. Although multivariable Cox regression adjusted for known confounders, residual confounding cannot be excluded. Nonetheless, the consistent three-group pattern and reproducible associations with mortality across cohorts support the robustness and cross-setting generalizability of our findings. Future studies should incorporate additional biomarkers, such as lactate, CRP, and pathogen-specific data, to refine trajectory-based phenotyping. Moreover, microbiological details, including resistance profiles and toxin gene expression, were unavailable; thus, we could not assess pathogen-driven modulation of OD or treatment response. More recently, SOFA-2 has been proposed to better reflect contemporary ICU practices (Ranzani et al., 2025). The present study was designed and conducted using the original SOFA score due to data availability and consistency across cohorts, which also facilitates comparison with prior trajectory-based studies. Future work will be needed to evaluate whether SOFA-2–based trajectories provide additional or incremental prognostic value in patients with SA-BSI. Despite these limitations, our study provides novel insight into the dynamic patterns of OD in SA-BSI and highlights the potential of trajectory-informed and interpretable ML approaches to enhance individualized risk assessment in critical care.

## Conclusions

In conclusion, this study identified three distinct SOFA score trajectory groups in ICU patients with SA-BSI, each representing unique patterns of OD progression and associated with markedly different clinical outcomes. Higher-risk trajectories, particularly Group 3, were characterized by severe and sustained cardiovascular compromise, which emerged as a prominent prognostic contributor across cohorts. Trajectory groups were also associated with meaningful clinical and biological gradients, supporting their phenotypic relevance. Moreover, we demonstrated that ML models based on early ICU admission features can accurately predict OD trajectories, and SHAP-based interpretation provided clinically meaningful insights into key physiological drivers. Together, these findings highlight the potential of trajectory-informed phenotyping for early risk stratification in critically ill patients with SA-BSI, complementing conventional severity scoring systems. Further prospective, multicenter studies are warranted to validate these findings, and future integration with microbiological profiling may enhance infection-specific precision care and antimicrobial stewardship in ICU settings.

## Data Availability

The MIMIC cohort analyzed in this study is derived from the MIMIC-IV database, which is publicly available through PhysioNet (https://mimic.mit.edu/). Access to the MIMIC-IV database requires completion of the required training and acceptance of the data use agreement on the PhysioNet website. Due to ethical and legal restrictions, the SAHZU cohort data are not publicly available but may be obtained from the corresponding author upon reasonable request.
