# Supplementary material for: Longitudinal SOFA score trajectories and risk stratification in ICU patients with Staphylococcus aureus bloodstream infection: insights from group-based trajectory modeling
Source: Front Cell Infect Microbiol. 2026 Feb 9;16:1756348. doi: 10.3389/fcimb.2026.1756348 (PMC12926426; doi:10.3389/fcimb.2026.1756348)
Supplement: Supplementary file 1 [file Table1.docx]

**Supplementary Methods**

**Data Sources and Ethical Approval**

This study was based on two independent cohorts. The first cohort was derived from the Medical Information Mart for Intensive Care IV (MIMIC-IV, version 3.0) database, which contains deidentified health data from patients admitted to the emergency department (ED) and intensive care units (ICUs) of Beth Israel Deaconess Medical Center (BIDMC), Boston, USA. Use of the database was approved by the institutional review board (IRB) of BIDMC, and a waiver of informed consent was granted due to data deidentification. The dataset includes demographic information, diagnoses, vital signs, and laboratory results for over 65,000 ICU patients and over 200,000 ED patients between 2008 and 2022 [1,2].

The second cohort comprised ICU-admitted patients from the Second Affiliated Hospital of Zhejiang University School of Medicine (SAHZU), a 3,200-bed tertiary hospital in Hangzhou, China, between January 2013 and June 2024. Clinical data were extracted from the hospital’s electronic medical record system (EMRS) with ethical approval from the institutional Ethics Committee (Approval No. 2024-0983). The committee waived the requirement for informed consent due to the retrospective nature of the study and complete removal of personally identifiable information.

For the MIMIC cohort, author Chen accessed the database after completing the Collaborative Institutional Training Initiative (CITI) program (Record ID: 65302480) and extracted data using PostgreSQL. SAHZU data were collected using structured queries within the EMRS and verified by clinical investigators.

**Clinical Definitions and Variable Collection**

*S. aureus* was classified as a nonpathogenic organism if it was cultured from only one of two blood culture bottles in a bilateral blood culture set, with culture time exceeding 48 hours [3].

Clinical variables were categorized as follows:

- Demographics: age, sex, race (MIMIC only), body mass index (BMI).
- Comorbidities: extracted using International Classification of Diseases (ICD)-9 and ICD-10 codes (**Supplementary Table S1**).
- Pre-infection risk factors: invasive mechanical ventilation, renal replacement therapy (RRT), central venous catheter (CVC) or peripherally inserted central catheter (PICC), and recent invasive procedures.
- Infection-related variables: methicillin resistance (MRSA), nosocomial origin, neutropenia.
- Treatment and monitoring data: timing of interventions, survival time, hospital and ICU length of stay.
- Organ dysfunction (OD) and inflammatory status: total and component Sequential Organ Failure Assessment (SOFA) scores; in SAHZU only, arterial blood gas results, C-reactive protein (CRP), and procalcitonin (PCT) levels were also included.

Operational definitions used in variable extraction included:

- Neutropenia: absolute neutrophil count ≤1,000/mm³.
- Circulatory disease: including myocardial infarction, congestive heart failure, and peripheral vascular disease.
- Malignancy: any malignant tumor, including lymphoma and leukemia, excluding skin malignancies.
- Invasive ventilation: defined as endotracheal or tracheostomy-based positive pressure ventilation, with or without tracheostomy procedure codes.

**SOFA Score Collection Strategy**

SOFA scores were extracted for five consecutive days surrounding *S. aureus* bloodstream infection (SA-BSI) onset: from 1 day before to 3 days after the first positive blood culture (i.e., Days -1, 0, +1, +2, +3). According to a prior study conducted by Gu et al. [4], which investigated different trajectories of inflammatory markers and vital signs in adults with suspected BSI, and indicated that the CRP of SA-BSI patients typically peaks on day 1 after the onset of BSI. Based on these findings and the fact that blood culture results typically take 3–5 days to finalize, we finally chose this window.

Due to the retrospective nature of the data and varying availability of laboratory and physiological measurements across time points, not all patients had complete daily SOFA scores for all five days. However, group-based trajectory modeling (GBTM), described below, accommodates longitudinal missingness through maximum likelihood estimation (MLE), which assumes that missing data are missing at random (MAR).

Therefore, no data imputation or forward-filling was performed for SOFA scores or subscores. All available data were included in the model estimation process, and patients with at least two SOFA measurements within the observation window were eligible for trajectory analysis.

**Group-Based Trajectory Modeling**

To identify distinct patterns of OD progression over time, GBTM was applied to the daily total SOFA scores from both cohorts.

Model development was performed using SOFA scores over 5 consecutive days (Days -1 to +3) in the MIMIC cohort. We fitted models with 1 to 5 trajectory groups and tested various polynomial forms (linear, quadratic, and cubic) for each group. Model selection was based on both statistical fit and clinical interpretability. The best-fitting model was chosen according to the following criteria:

- Bayesian Information Criterion (BIC) and Akaike Information Criterion (AIC): lower values indicate better fit with penalization for complexity;
- Average Posterior Probability (AvePP): ≥0.70 considered acceptable classification reliability;
- Entropy: higher values (closer to 1.0) reflect better separation between groups;
- Subgroup Proportions: all groups required to include ≥5% of the cohort;
- Odds of Correct Classification (OCC): values >5.0 indicate strong group assignment confidence.

After selecting the final model, each patient was assigned to the trajectory group with the highest posterior probability. The model was implemented using a MLE framework, which accommodates missing values under the MAR assumption.

Model validation was performed by applying the MIMIC-derived model to the external SAHZU cohort, assigning patients using the same parameter estimates and classification rules. Group distribution and survival associations in the SAHZU cohort were compared to MIMIC for validation consistency.

**Statistical Analysis and Software**

Cox proportional hazards models were used to evaluate the association between SOFA trajectory groups and in-hospital mortality. Covariates were selected based on clinical relevance and statistical significance (*P* < 0.05) in univariable analysis. Variables retained in multivariable models were those with *P* < 0.05 after adjustment. Trajectory Group 1 (representing the lowest and most stable SOFA scores) was used as the reference category. Kaplan–Meier survival curves were generated to compare 28-day and 90-day mortality between trajectory groups, and statistical differences were assessed using the log-rank test.

All statistical analyses were performed using the following software packages:

- R software (version 4.4.2; Lucent Technologies, USA), including packages survival and rms;
- Python (version 3.10; Python Software Foundation, USA), with packages including pandas, numpy, scikit-learn, and matplotlib for data manipulation and visualization;
- Zstats (version 1.0; www.zstats.net) for reclassification statistics;
- SPSS (version 26.0; IBM Corp., Armonk, NY, USA) for data preprocessing;
- Stata/MP (version 17; StataCorp, College Station, TX, USA) for figure generation and statistical modeling;
- Microsoft Excel (Microsoft Corp., Redmond, WA, USA) for table formatting and quality checks.

Continuous variables were presented as mean ± standard deviation (SD) or median [interquartile range, IQR], depending on distribution. Categorical variables were expressed as percentages. Between-group comparisons were conducted using Student’s t-test or Mann–Whitney U test (for continuous variables) and Pearson’s chi-squared or Fisher’s exact test (for categorical variables). A two-sided *P*-value < 0.05 was considered statistically significant.

**Supplementary Results**

**GBTM classification performance and posterior probability validation**

GBTM was applied to daily total SOFA scores from Days –1 to +3 in the MIMIC cohort. Candidate models with one to five trajectory groups were tested using different polynomial functions (linear, quadratic, and cubic). Model selection was guided by multiple statistical and clinical considerations. Although the five-group model showed the best statistical fit (i.e., lowest AIC and BIC), it produced a subgroup comprising only 3.6% of patients, indicating potential overfitting. Similarly, the four-group model included a group with only 6.6% membership and demonstrated a notable drop in entropy (0.787), suggesting weakened classification certainty. In contrast, the three-group model showed strong classification performance with a high entropy of 0.829 and balanced group sizes (48.2%, 39.9%, 11.9%), supporting both statistical robustness and clinical interpretability. All candidate models had AvePP >0.70 and OCC >5.0, indicating generally reliable classification across models (**Supplementary Table S2**). Ultimately, the three-group model was selected as optimal.

The SAHZU cohort was labeled as “out of sample (OOS)” when modeling the MIMIC cohort; therefore, the most probable trajectory groups and posterior probabilities of each trajectory group for each patient in the SAHZU cohort were determined by the same model derived from the MIMIC cohort. And the classification remained robust. Among 151 SAHZU patients, 139 (92.1%) had a maximum posterior probability (maxPP) > 0.7, and all patients had maxPP > 0.5. The final AvePPs were 0.93, 0.90, and 0.94 for Groups 1 to 3, respectively, confirming acceptable trajectory assignment accuracy in the validation set.

**Pairwise Survival Differences**

Kaplan-Meier curves for 90-day mortality (**Supplementary Figure S3**) showed clear separation between trajectory groups in both cohorts. As detailed in **Supplementary Table S6**, comparisons between Group 1 vs. Group 3 and Group 2 vs. Group 3 were statistically significant (log-rank *p* < 0.001) in both cohorts. However, the survival difference between Group 1 and Group 2 reached statistical significance only in the MIMIC cohort. This discrepancy may be attributed to limited sample size or cohort-specific clinical practices in the SAHZU population.

**SOFA Score Completeness**

**Supplementary Table S7** summarizes the availability of daily SOFA scores across the five observation days. In the MIMIC cohort (n = 834), the number of patients with available total SOFA scores on Days –1, 0, +1, +2, and +3 were 169 (20.3%), 561 (67.3%), 732 (87.8%), 670 (80.3%), and 549 (65.8%), respectively. In contrast, SOFA score completeness in the SAHZU cohort (n = 151) exceeded 75% across all time points.

No imputation was performed, as the GBTM framework inherently accommodates missing longitudinal data through MLE, assuming MAR. Given the high availability of SOFA data during the early peak inflammatory phase (Days 0–2), and the non-monotonic missingness pattern consistent with real-world ICU data collection, no systematic bias was anticipated.

**Supplementary Discussion**

An additional insight from our trajectory analysis was the divergent SOFA progression patterns between Groups 2 and 3. Group 2 exhibited a steady, monotonic increase in SOFA scores, while Group 3 peaked early at Day 2 before declining slightly, albeit remaining within a critically elevated range. Despite this apparent improvement, Group 3 had significantly worse outcomes. This suggests that short-term decreases in SOFA scores do not necessarily indicate recovery and may obscure persistent OD or ongoing systemic inflammation—key drivers of mortality in SA-BSI [5,6].These observations underscore the limitations of relying solely on static or short-interval SOFA changes to guide prognosis.

This study also offers several notable strengths: i) Novel Application in SA-BSI: To our knowledge, this is among the first studies to apply GBTM to analyze SOFA trajectories in SA-BSI patients. This method captures dynamic OD patterns, providing a more nuanced understanding of disease progression compared to static severity scores.; ⅱ) Validation Across Independent Cohorts: The use of two geographically and structurally distinct ICU cohorts (MIMIC and SAHZU) enhances the generalizability of our findings. Despite differences in clinical practices, biomarker availability, and patient profiles, the trajectory model showed strong reproducibility and consistent prognostic value. ⅲ) Mechanistic and Clinical Insight: The consistent identification of cardiovascular dysfunction as the dominant contributor to poor prognosis in Group 3 offers clinically actionable insight. This supports the use of trajectory-informed monitoring to guide early escalation of cardiovascular support and prioritize high-risk patients for more intensive care. Together, these supplementary findings reinforce the central message of the main manuscript: trajectory-informed phenotyping adds clinical value to early ICU risk stratification in SA-BSI, and can support decision-making during the critical diagnostic window before microbiological confirmation. These results further strengthen the case for integrating trajectory-based models into dynamic ICU prognostic workflows.

**Abbreviations**

MIMIC, Medical Information Mart for Intensive Care; ED, emergency department; ICU, intensive care unit; BIDMC, the Beth Israel Deaconess Medical Center; IRB, institutional review board; SAHZU, the Second Affiliated Hospital of Zhejiang University School of Medicine; EMRS, electronic medical record system; CITI, the Collaborative Institutional Training Initiative; BMI, body mass index; ICD, International Classification of Diseases; RRT, renal replacement therapy; CVC, central venous catheter; PICC, peripherally inserted central venous catheters; MRSA, Methicillin-resistant Staphylococcus aureus; OD, organ dysfunction; SOFA, sequential organ failure assessment; CRP, C-reactive protein; PCT, procalcitonin; SA-BSI, Staphylococcus aureus bloodstream infection; GBTM, group-based trajectory modeling; MLE, maximum likelihood estimation; MAR, missing at random; BIC, Bayesian Information Criterion; AIC, Akaike Information Criterion; AvePP, Average Posterior Probability; OCC, Odds of Correct Classification; SD, standard deviation; IQR, interquartile range; OOS, out of sample; maxPP, maximum posterior probability.

**References:**

1. Xu H, Liu Y, Niu H, Wang H, Zhan F. Impact of body mass index on mortality outcomes in intensive care patients with Staphylococcus aureus sepsis: A retrospective analysis. Magira E, editor. PLOS ONE. 2024 Aug 6;19(8):e0308471.

2. Johnson AEW, Bulgarelli L, Shen L, Gayles A, Shammout A, Horng S, et al. MIMIC-IV, a freely accessible electronic health record dataset. Sci Data. 2023 Jan 3;10(1):1.

3. Zheng C, Zhang S, Chen Q, Zhong L, Huang T, Zhang X, et al. Clinical characteristics and risk factors of polymicrobial Staphylococcus aureus bloodstream infections. Antimicrob Resist Infect Control. 2020 May 27;9(1):76.

4. Gu Q, Wei J, Yoon CH, Yuan K, Jones N, Brent A, et al. Distinct patterns of vital sign and inflammatory marker responses in adults with suspected bloodstream infection. J Infect. 2024 May;88(5):106156.

5. Zorowitz RD. ICU-Acquired Weakness: A Rehabilitation Perspective of Diagnosis, Treatment, and Functional Management. Chest. 2016 Oct;150(4):966–71.

6. Minne L, Abu-Hanna A, de Jonge E. Evaluation of SOFA-based models for predicting mortality in the ICU: A systematic review. Crit Care. 2008 Dec 17;12(6):R161.

**Supplementary Table S1. ICD code for comorbidities.**

| Comorbidities | ICD-9 | ICD-10 |
| --- | --- | --- |
| Hypertension | '401', '405', '642', '99791' | 'I10', 'I15', 'O10', 'O11', 'O13', 'O16', 'P292' |
| Diabetes | '2500' - '2509' | 'E100' - 'E149' |
| Chronic pulmonary disease | '4168', '4169', '490' - '505', '5064', '5081', '5088' | 'I278', 'I279', 'J40' - 'J47', 'J60' - 'J67', 'J684', 'J701', 'J703' |
| Circulation diseases | '0930', '39891', '40201', '40211', '40291', '40401', '40403', '40411', '40413', '40491', '40493', '410', '412', '4254' - '4259', '428', '4373', '440', '441', '4431' - '4439', '4471', '5571', '5579', 'V434' | 'I099', 'I110', 'I130', 'I132', 'I21', 'I22', 'I252', 'I255', 'I420', 'I425' - 'I429', 'I43', 'I50', 'I70', 'I71', 'I731', 'I738', 'I739', 'I771', 'I790', 'I792', 'K551', 'K558', 'K559', 'P290', 'Z958', 'Z959' |
| Cerebrovascular disease | '36234', '430', '438' | 'G45', 'G46', 'H340', 'I60', 'I69' |
| Renal disease | '40301', '40311', '40391', '40402', '40403', '40412', '40413', '40492', '40493', '582', '5830' - '5837', '585', '586', '5880', 'V420', 'V451', 'V56' | 'I120', 'I131', 'N032' - 'N037', 'N052' - 'N057', 'N18', 'N19', 'N250', 'Z490' - 'Z492', 'Z940', 'Z992' |
| Liver disease | '07022', '07023', '07032', '07033', '07044', '07054', '0706', '0709', '4560' - '4562', '570', '571', '5722' - '5728', '5733', '5734', '5738', '5739', 'V427' | 'B18', 'I850', 'I859', 'I864', 'I982', 'K700' - 'K704', 'K709', 'K711', 'K713' - 'K715', 'K717', 'K721', 'K729', 'K73', 'K74', 'K760', 'K762' - 'K769', 'Z944' |
| Malignant cancer | '140' - '172', '1740' - '1958', '200' - '208', '2386' | 'C43', 'C88', 'C00' - 'C26', 'C30' - 'C34', 'C37' - 'C41', 'C45' - 'C58', 'C60' - 'C76', 'C81' - 'C85', 'C90' - 'C97' |

**Supplementary Table S2. Fit statistics for different numbers of trajectory groups.**

| Trajectory groups | Loglik | AIC | BIC(N=2681) | BIC(N=834) | Entropy | Group membership (%) | AvePP (per trajectory group) | OCC (per trajectory group) |
| --- | --- | --- | --- | --- | --- | --- | --- | --- |
| 1 | -7316.95 | -7320.95 | -7332.73 | -7330.40 | - | 100 | 1.00 | - |
| 2 | -6745.04 | -6753.04 | -6776.61 | -6771.94 | 0.833 | 70.17/29.83 | 0.96/0.93 | 10.08/33.18 |
| **3** | **-6493.62** | **-6506.62** | **-6544.93** | **-6537.34** | **0.829** | **48.22/39.88/11.89** | **0.93/0.90/0.94** | **15.02/13.38/103.52** |
| 4 | -6405.36 | -6421.36 | -6468.51 | -6459.17 | 0.787 | 34.26/38.98/20.17/6.59 | 0.89/0.85/0.88/0.94 | 15.02/8.65/31.43/185.60 |
| 5 | -6364.18 | -6384.18 | -6443.12 | -6431.44 | 0.758 | 24.43/34.00/27.76/12.22/3.60 | 0.82/0.80/0.84/0.91/0.90 | 15.53/7.80/13.96/78.81/207.18 |

Loglik, log likelihood; AIC, Akaike Information Criterion; BIC, Bayesian Information Criterion; AvePP, the average posterior probability for each group; OCC, odds of correct classification.

**Supplementary Table S3. Additional collected factors for the SAHZU cohort.**

| Factor | Total (n = 151) | 1 (n = 75) | 2 (n = 53) | 3 (n = 23) | Statistic | P |
| --- | --- | --- | --- | --- | --- | --- |
| CRP, mg/L, IQR | 108.70 (56.98, 178.03) | 82.55 (44.12,139.75) | 131.50 (68.40,181.60) | 200.80 (73.55,252.90) | χ²=13.73# | **0.001** |
| PCT, ng/ml, IQR | 0.78 (0.18, 3.28) | 0.28 (0.13,1.12) | 1.25 (0.42,5.87) | 7.47 (0.97,14.75) | χ²=29.66# | **<.001** |
| APACHE II, IQR | 15.00 (12.00, 21.00) | 14.00 (10.50,18.00) | 18.00 (15.00,23.00) | 19.00 (15.00,24.50) | χ²=22.79# | **<.001** |
| Smoker, n(%) | 56 (37.09) | 29 (38.67) | 19 (35.85) | 8 (34.78) | χ²=0.17 | 0.920 |
| Comorbidities |  |  |  |  |  |  |
| Burn, n(%) | 21 (13.91) | 15 (20.00) | 4 (7.55) | 2 (8.70) | χ²=4.64 | 0.098 |
| Trauma, n(%) | 37 (24.50) | 21 (28.00) | 13 (24.53) | 3 (13.04) | χ²=2.13 | 0.345 |
| Risk factors |  |  |  |  |  |  |
| Blood transfusion, n(%) | 16 (10.60) | 6 (8.00) | 6 (11.32) | 4 (17.39) | χ²=1.68 | 0.431 |
| ECMO, n(%) | 1 (0.66) | 0 (0.00) | 0 (0.00) | 1 (4.35) | - | 0.152 |
| Immunosuppressants, n(%) | 3 (1.99) | 1 (1.33) | 2 (3.77) | 0 (0.00) | - | 0.739 |
| Urinary catheter, n(%) | 126 (83.44) | 66 (88.00) | 40 (75.47) | 20 (86.96) | χ²=3.77 | 0.152 |
| Drainage Tube, n(%) | 69 (45.70) | 35 (46.67) | 24 (45.28) | 10 (43.48) | χ²=0.08 | 0.962 |
| Neutropenia, n(%) | 3 (1.99) | 1 (1.33) | 1 (1.89) | 1 (4.35) | - | 0.555 |
| Treatments after BSI onset |  |  |  |  |  |  |
| ECMO, n(%) | 4 (2.65) | 1 (1.33) | 1 (1.89) | 2 (8.70) | - | 0.150 |
| Chemistry |  |  |  |  |  |  |
| Albumin, g/L, Mean ± SD | 30.15 (26.22, 34.30) | 31.40 (26.15,36.15) | 29.35 (26.30,32.80) | 29.90 (26.75,34.25) | χ²=1.80# | 0.407 |
| Coagulation |  |  |  |  |  |  |
| D-dimmer, Mean ± SD | 3630.00 (2180.00, 6550.00) | 2740.00 (1637.50,4342.50) | 4740.00 (2305.00,10692.50) | 6170.00 (3440.00,8220.00) | χ²=19.06# | **<.001** |
| Arterial blood gas |  |  |  |  |  |  |
| PH, IQR | 7.43 (7.39, 7.48) | 7.45 (7.41,7.49) | 7.43 (7.39,7.47) | 7.37 (7.34,7.45) | χ²=10.01# | **0.007** |
| P/F, IQR | 256.67 (198.36, 337.00) | 291.43 (212.50,359.82) | 256.67 (210.75,332.07) | 184.00 (146.90,245.62) | χ²=16.96# | **<.001** |
| Lactate, mmol/L, IQR | 1.79 (1.20, 2.70) | 1.50 (1.10,2.30) | 1.70 (1.40,2.50) | 4.10 (2.10,5.55) | χ²=23.79# | **<.001** |

IQR, interquartile range; ECMO, Extracorporeal Membrane Oxygenation; BSI, bloodstream infection; SD, standard deviation; P/F partial pressure arterial oxygen and fraction of inspired oxygen; CRP, C-reactive protein; PCT, procalcitonin; APACHE, acute physiology and chronic health evaluation.

**Supplementary Table S4. Cox univariate and multivariate regression of in-hospital mortality for training cohort (MIMIC-IV) and validation cohort (SAHZU).**

| Variables | Training cohort (MIMIC-IV) | | | | | | | | | | |  | Validation cohort (SAHZU) | | | | | | | | | | |
| --- | --- | --- | --- | --- | --- | --- | --- | --- | --- | --- | --- | --- | --- | --- | --- | --- | --- | --- | --- | --- | --- | --- | --- |
|  | Univariable | | | | |  | Multivariable | | | | |  | Univariable | | | | |  | Multivariable | | | | |
|  | β | S.E | Z | *P* | HR (95%CI) |  | β | S.E | Z | *P* | HR (95%CI) |  | β | S.E | Z | *P* | HR (95%CI) |  | β | S.E | Z | *P* | HR (95%CI) |
| Group |  |  |  |  |  |  |  |  |  |  |  |  |  |  |  |  |  |  |  |  |  |  |  |
| 1 |  |  |  |  | 1.00 (Reference) |  |  |  |  |  | 1.00 (Reference) |  |  |  |  |  | 1.00 (Reference) |  |  |  |  |  | 1.00 (Reference) |
| 2 | 0.61 | 0.10 | 5.96 | **<.001** | 1.83 (1.50 ~ 2.24) |  | 0.36 | 0.16 | 2.19 | **0.029** | 1.43 (1.04 ~ 1.97) |  | 0.51 | 0.28 | 1.79 | 0.073 | 1.66 (0.95 ~ 2.91) |  | 0.37 | 0.38 | 0.97 | 0.333 | 1.44 (0.69 ~ 3.03) |
| 3 | 1.28 | 0.14 | 9.30 | **<.001** | 3.59 (2.74 ~ 4.70) |  | 1.50 | 0.29 | 5.22 | **<.001** | 4.49 (2.56 ~ 7.90) |  | 1.60 | 0.31 | 5.16 | **<.001** | 4.94 (2.70 ~ 9.07) |  | 1.48 | 0.65 | 2.27 | **0.023** | 4.38 (1.22 ~ 15.71) |
| Age | 0.03 | 0.00 | 10.59 | **<.001** | 1.03 (1.03 ~ 1.04) |  | 0.03 | 0.00 | 7.47 | **<.001** | 1.03 (1.02 ~ 1.04) |  | 0.01 | 0.01 | 1.23 | 0.220 | 1.01 (0.99 ~ 1.02) |  |  |  |  |  |  |
| Male | 0.20 | 0.10 | 2.11 | **0.035** | 1.22 (1.01 ~ 1.48) |  | 0.16 | 0.12 | 1.33 | 0.183 | 1.17 (0.93 ~ 1.49) |  | -0.01 | 0.28 | -0.02 | 0.985 | 0.99 (0.58 ~ 1.71) |  |  |  |  |  |  |
| Hypertension | -0.22 | 0.10 | -2.22 | **0.026** | 0.80 (0.66 ~ 0.97) |  | 0.09 | 0.16 | 0.55 | 0.581 | 1.09 (0.80 ~ 1.48) |  | 0.15 | 0.25 | 0.61 | 0.544 | 1.16 (0.71 ~ 1.90) |  |  |  |  |  |  |
| Diabetes | 0.30 | 0.09 | 3.18 | **0.001** | 1.35 (1.12 ~ 1.62) |  | 0.08 | 0.12 | 0.67 | 0.505 | 1.09 (0.85 ~ 1.39) |  | 0.53 | 0.30 | 1.76 | 0.079 | 1.70 (0.94 ~ 3.06) |  |  |  |  |  |  |
| Chronic pulmonary disease | 0.23 | 0.10 | 2.28 | **0.023** | 1.26 (1.03 ~ 1.54) |  | -0.08 | 0.13 | -0.60 | 0.548 | 0.92 (0.71 ~ 1.20) |  | 0.30 | 0.60 | 0.51 | 0.610 | 1.36 (0.42 ~ 4.35) |  |  |  |  |  |  |
| Circulation diseases | 0.76 | 0.09 | 8.02 | **<.001** | 2.14 (1.77 ~ 2.57) |  | 0.64 | 0.13 | 4.84 | **<.001** | 1.90 (1.46 ~ 2.46) |  | -0.39 | 0.41 | -0.96 | 0.337 | 0.68 (0.31 ~ 1.50) |  |  |  |  |  |  |
| Cerebrovascular disease | 0.32 | 0.12 | 2.73 | **0.006** | 1.37 (1.09 ~ 1.72) |  | 0.43 | 0.15 | 2.85 | **0.004** | 1.54 (1.14 ~ 2.07) |  | -0.29 | 0.31 | -0.95 | 0.342 | 0.75 (0.41 ~ 1.37) |  |  |  |  |  |  |
| Renal disease | 0.55 | 0.09 | 5.86 | **<.001** | 1.73 (1.44 ~ 2.07) |  | 0.04 | 0.15 | 0.25 | 0.801 | 1.04 (0.77 ~ 1.41) |  | 0.26 | 1.01 | 0.25 | 0.800 | 1.29 (0.18 ~ 9.38) |  |  |  |  |  |  |
| Liver disease | 0.08 | 0.11 | 0.73 | 0.466 | 1.09 (0.87 ~ 1.35) |  |  |  |  |  |  |  | 0.88 | 0.47 | 1.88 | 0.060 | 2.40 (0.96 ~ 5.98) |  |  |  |  |  |  |
| Malignant cancer | 0.88 | 0.13 | 6.89 | **<.001** | 2.42 (1.88 ~ 3.11) |  | 0.80 | 0.18 | 4.43 | **<.001** | 2.22 (1.56 ~ 3.17) |  | 0.31 | 0.52 | 0.60 | 0.551 | 1.36 (0.49 ~ 3.74) |  |  |  |  |  |  |
| Invasive ventilation | 0.17 | 0.14 | 1.22 | 0.222 | 1.19 (0.90 ~ 1.57) |  |  |  |  |  |  |  | 0.62 | 0.34 | 1.80 | 0.072 | 1.85 (0.95 ~ 3.64) |  |  |  |  |  |  |
| Invasive procedure | 0.00 | 0.09 | 0.01 | 0.990 | 1.00 (0.83 ~ 1.20) |  |  |  |  |  |  |  | 0.23 | 0.27 | 0.85 | 0.397 | 1.25 (0.74 ~ 2.11) |  |  |  |  |  |  |
| RRT | 0.44 | 0.34 | 1.31 | 0.192 | 1.55 (0.80 ~ 3.00) |  |  |  |  |  |  |  | 0.21 | 0.47 | 0.46 | 0.644 | 1.24 (0.50 ~ 3.09) |  |  |  |  |  |  |
| CVC/PICC | 0.51 | 0.23 | 2.23 | **0.026** | 1.66 (1.06 ~ 2.61) |  | 0.50 | 0.24 | 2.08 | **0.037** | 1.65 (1.03 ~ 2.64) |  | 0.26 | 0.33 | 0.79 | 0.429 | 1.30 (0.68 ~ 2.47) |  |  |  |  |  |  |
| MRSA | 0.26 | 0.11 | 2.44 | **0.015** | 1.29 (1.05 ~ 1.59) |  | 0.07 | 0.13 | 0.54 | 0.587 | 1.07 (0.83 ~ 1.39) |  | 0.05 | 0.31 | 0.18 | 0.861 | 1.06 (0.58 ~ 1.94) |  |  |  |  |  |  |
| Nosocomial infection | 0.22 | 0.11 | 2.02 | **0.043** | 1.25 (1.01 ~ 1.55) |  | -0.15 | 0.14 | -1.06 | 0.288 | 0.86 (0.66 ~ 1.13) |  | -0.01 | 0.26 | -0.05 | 0.960 | 0.99 (0.60 ~ 1.63) |  |  |  |  |  |  |
| SOFA | 0.09 | 0.01 | 6.23 | **<.001** | 1.09 (1.06 ~ 1.12) |  | 0.00 | 0.03 | 0.08 | 0.940 | 1.00 (0.95 ~ 1.05) |  | 0.14 | 0.03 | 4.61 | **<.001** | 1.15 (1.08 ~ 1.22) |  | -0.00 | 0.07 | -0.01 | 0.989 | 1.00 (0.88 ~ 1.14) |

HR, Hazard Ratio, CI, Confidence Interval; RRT, renal replacement therapy; CVC, central venous catheter; PICC, peripherally inserted central venous catheters; MRSA, Methicillin-resistant Staphylococcus aureus.

**Supplementary Table S5. Unadjusted and adjusted Cox regression for the training cohort (MIMIC-IV) and validation cohort (SAHZU).**

| Variables | Training cohort (MIMIC-IV) | | | | |  | Validation cohort (SAHZU) | | | | |
| --- | --- | --- | --- | --- | --- | --- | --- | --- | --- | --- | --- |
|  | Unadjusted | |  | Adjusted | |  | Unadjusted | |  | Adjusted | |
|  | HR (95%CI) | *P* |  | HR (95%CI) | *P* |  | HR (95%CI) | *P* |  | HR (95%CI) | *P* |
| Group |  |  |  |  |  |  |  |  |  |  |  |
| 1 | 1.00 (Reference) |  |  | 1.00 (Reference) |  |  | 1.00 (Reference) |  |  | 1.00 (Reference) |  |
| 2 | 1.83 (1.50 ~ 2.24) | **<.001** |  | 1.56 (1.27 ~ 1.91) | **<.001** |  | 1.66 (0.95 ~ 2.91) | 0.073 |  | 1.59 (0.89 ~ 2.83) | 0.117 |
| 3 | 3.59 (2.74 ~ 4.70) | **<.001** |  | 4.60 (3.49 ~ 6.07) | **<.001** |  | 4.94 (2.70 ~ 9.07) | **<.001** |  | 5.36 (2.87 ~ 10.00) | **<.001** |

Adjusted: Age, Circulation diseases, Cerebrovascular disease, malignant cancer, CVC/PICC. HR: Hazard Ratio, CI: Confidence Interval.

**Supplementary Table S6. Pairwise comparisons between groups using the Log-Rank test for the training cohort (MIMIC-IV) and validation cohort (SAHZU).**

| Group | Training cohort (MIMIC-IV) | | | | |  | Validation cohort (SAHZU) | | | | |
| --- | --- | --- | --- | --- | --- | --- | --- | --- | --- | --- | --- |
|  | 28-day mortality | |  | 90-day mortality | |  | 28-day mortality | |  | 90-day mortality | |
|  | 1 | 2 |  | 1 | 2 |  | 1 | 2 |  | 1 | 2 |
| 2 | **** | - |  | **** | - |  | + | - |  | + | - |
| 3 | **** | **** |  | **** | **** |  | **** | *** |  | **** | *** |

****: 0 < *p*-value < 0.0001; ***: 0.0001< *p*-value < 0.001; **: 0.001< *p*-value < 0.01; +: 0.05< *p*-value <0.1.

**Supplementary Table S7. Daily availability of total SOFA scores from Day –1 to Day +3.**

| **Cohort** | **Day -1** | **Day 0** | **Day 1** | **Day 2** | **Day 3** |
| --- | --- | --- | --- | --- | --- |
| MIMIC (n=834) | 169 (20.3%) | 561 (67.3%) | 732 (87.8%) | 670 (80.3%) | 549 (65.8%) |
| SAHZU (n=151) | 114 (75.5%) | 151 (100%) | 150 (99.3%) | 145 (96.0%) | 136 (90.1%) |


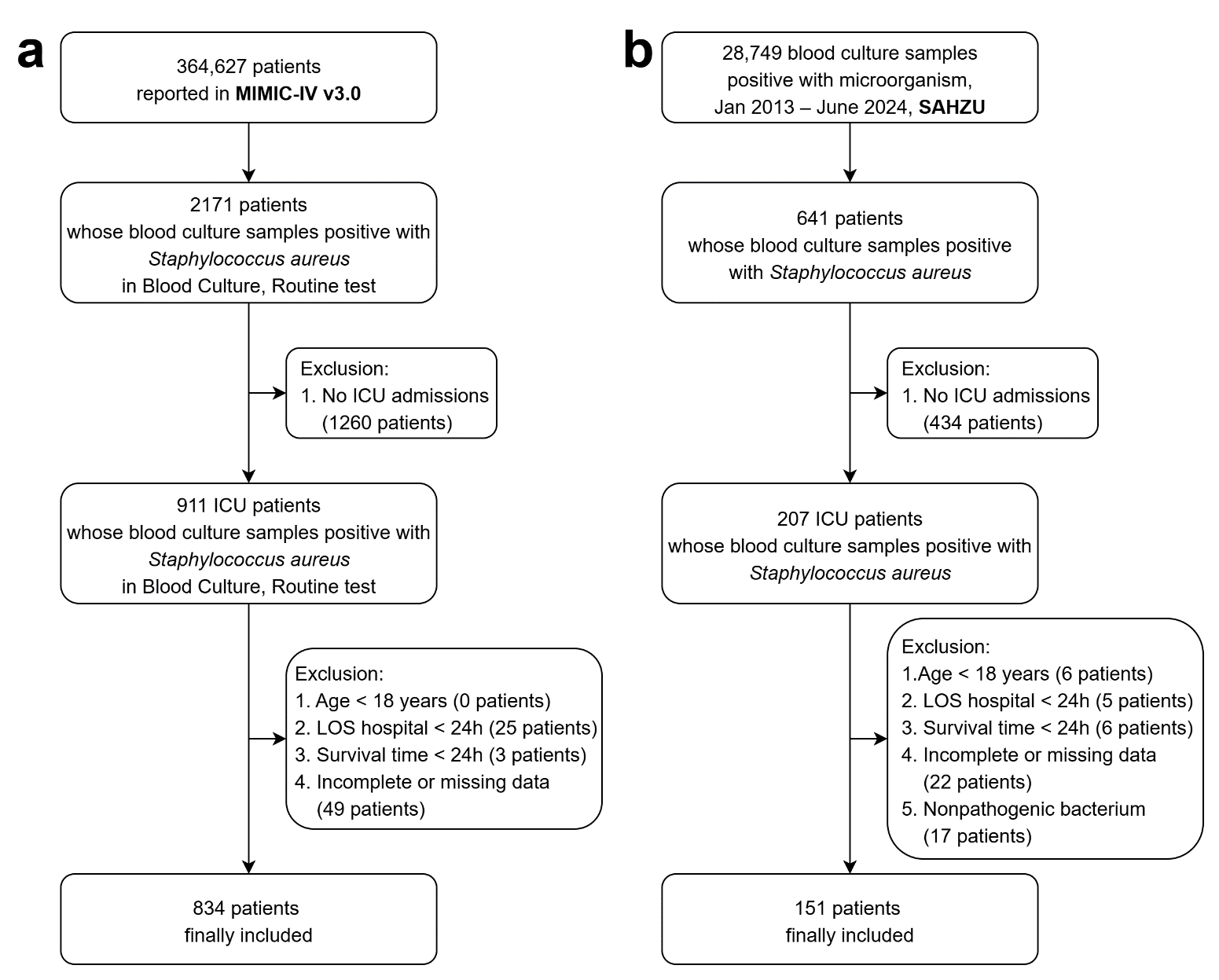


**Supplementary Figure S1. Flow chart of patient inclusion for the MIMIC-IV cohort (a) and the SAHZU cohort (b).** This diagram outlines the stepwise screening and exclusion process for ICU patients with positive blood cultures for *Staphylococcus aureus* in the MIMIC-IV **(a)** and SAHZU **(b)** cohorts. Exclusion criteria included non-ICU admissions, age under 18, short hospital or survival durations (<24 hours), incomplete data, and presence of nonpathogenic isolates (SAHZU only). Final analyses included 834 patients from MIMIC-IV and 151 from SAHZU. ICU, intensive care unit; LOS, length of stay.

**Supplementary Figure S2. Trajectories of SOFA subscores by trajectory group in the MIMIC cohort (a) and the SAHZU cohort (b).** Each panel displays the temporal progression of individual SOFA subscores (respiration, coagulation, liver, renal, CNS, cardiovascular) from Day –1 to Day 3 across the three trajectory groups identified by GBTM. Group 3 consistently demonstrated the highest and most rapidly increasing scores, particularly in the cardiovascular and respiratory domains. In contrast, Group 1 maintained low and stable subscores across all systems. CNS, central nervous system.

**Supplementary Figure S3. Kaplan–Meier curves for 90-day survival in the MIMIC cohort (a) and the SAHZU cohort (b), stratified by SOFA trajectory group.** Each survival curve represents one of the three organ dysfunction trajectory groups derived from GBTM. Patients in Group 3 consistently showed significantly worse long-term survival compared to Groups 1 and 2 (log-rank test, p < 0.0001). Number at risk is shown below each plot for each time point. The shaded areas represent 95% confidence intervals.
